# Testing the Integrated Motivational-Volitional Model of suicidal behavior in a young Danish population

**DOI:** 10.1186/s12888-026-08049-2

**Published:** 2026-04-10

**Authors:** Erik Christiansen, Agnieszka Konieczna, Christina Petrea Larsen, Sarah Grube Jakobsen, Rory C. O´Connor

**Affiliations:** 1 Centre for Suicide Research, Boder 28-30, st. th., Odense, 5000 Denmark; 2 Unit of Mental Health Research, Southwest Denmark, Department of Regional Health Services, University of Southern Denmark, Aabenraa, Denmark; 3 Department of Regional Health Research, University of Southern Denmark, Odense, Denmark; 4 Research Unit of Health Promotion, Department of Public Health, University of Southern Denmark, Esbjerg, Denmark; 5 Suicidal Behaviour Research Lab, School of Health & Wellbeing, University of Glasgow, Glasgow, Scotland

**Keywords:** Non-suicidal self-injury (NSSI), Suicide attempt, Self-harm, Integrated Motivational-Volitional model of suicidal behaviour (IMV-model), Ideation-to-action models, Youth

## Abstract

**Introduction:**

The Integrated Motivational-Volitional model for suicidal behaviour (IMV-model) is a predominant model of suicide risk which was developed to enhance understanding of the emergence of suicidal thoughts and the transition from suicidal thoughts to suicidal behaviour.

**Methods:**

This study aimed to test the central tenets of the IMV-model within a young Danish population (age 13–19 years) and to explore whether a selection of moderators was important in the context of intentional self-harm (NSSI and suicide attempts), NSSI or suicide attempts as outcome measures. Pathway analysis was conducted, using a large sample of Danish students.

**Results:**

Based on data from 1,581 individuals we found a strong fit for the central tenets of the IMV-model. We found evidence that threat-to-self and motivational moderators were important, but also that the volitional moderators (pain tolerance, fearlessness about death, impulsivity, mental images and being exposed to suicidal behavior) acted as moderators in all the models.

**Conclusion:**

The IMV model provides a strong framework for understanding intentional self-harm in youth, and this study offers some empirical support for the model. Next steps include applying the model to identify individuals at risk of suicidal ideation and behavior, using it as a foundation for developing youth-specific interventions, and conducting further tests of the model with more robust longitudinal epidemiological designs.

**Supplementary Information:**

The online version contains supplementary material available at 10.1186/s12888-026-08049-2.

## Introduction

Suicidal behaviour is a significant international public health problem. It is estimated that more than 700.000 individuals die by suicide and many more people attempt suicide each year. Suicide accounts for 1.4% of all worldwide deaths and it is the fourth leading cause of death among young people (15–29 years) (World Health Organization, [42]).

Over the last 50 years researchers have analysed the associations between risk factors and suicidal behaviour. The list of statistically significant risk factors is long, and spans social, financial, mental, and somatic factors [37]. Despite years of research the predictive clinical value of these risk factors remains low and is only slightly better than chance in predicting suicide deaths [8]. Such risk factors are still used in suicide risk assessments, but with poor outcome [3, 31].

More recently, ideation-to-action models have been developed to provide an alternative way of organizing and analysing risk/protective factors (such as the Interpersonal Theory, the Integrated Motivational-Volitional Model, the Three Step Theory and the Fluid Vulnerability Theory; see [14] for a review). It is hoped that these can result in a better understanding of the suicidal process including the transition from suicidal ideation to suicidal behaviour, and thus improve prevention efforts [23]. What each of these models have in common is the central premise that the factors associated with the development of suicidal ideation are distinct from those associated with the progression from suicidal ideation to attempts [14]. The models, however, differ in terms of the specific factors which they posit are associated with suicide risk.

**Fig. 1:**
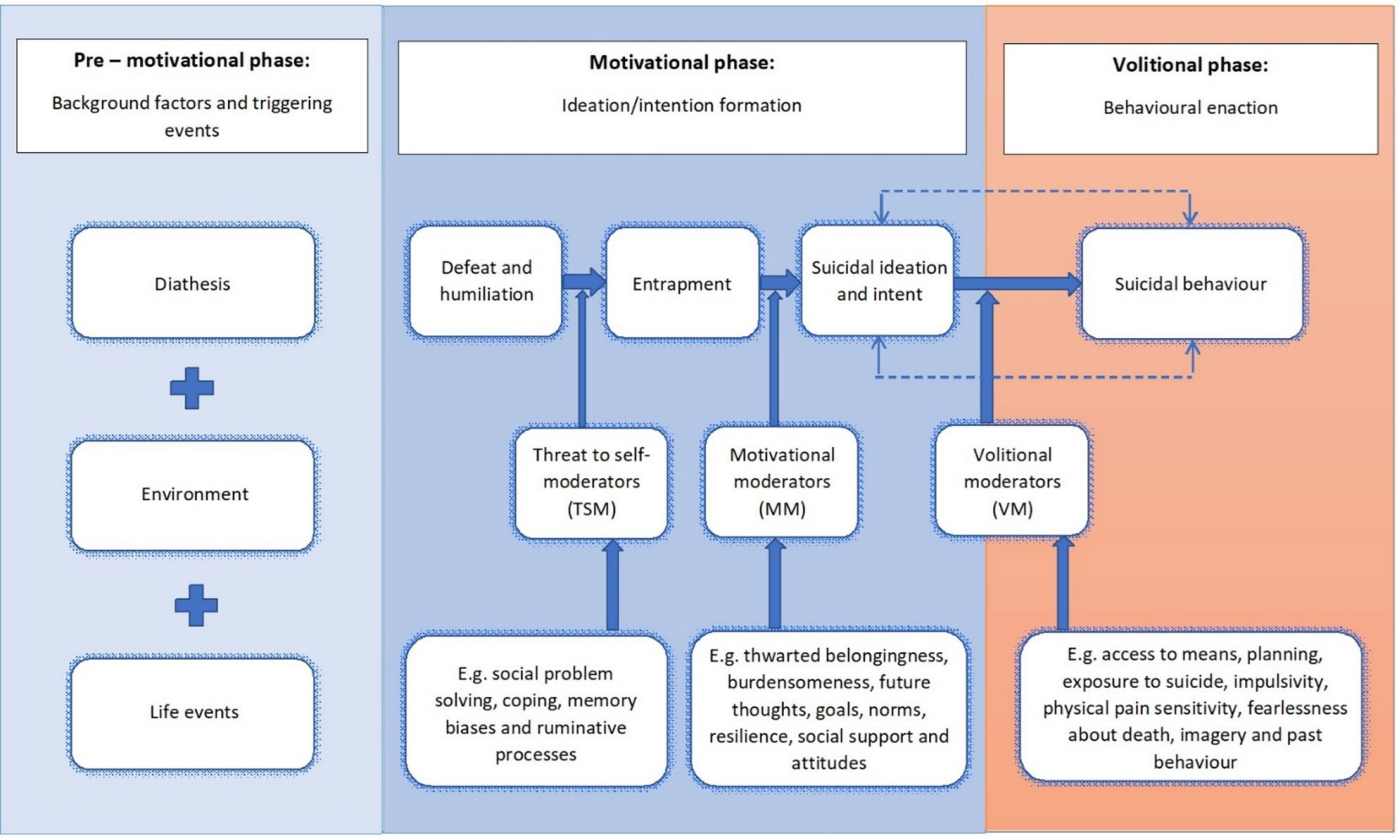
The integrated motivational-volitional model of suicidal behavior

In this study we focus on the Integrated Motivational-Volitional model of suicidal behaviour (IMV model; [22]) as it includes the vast majority of factors contained within the other models. The IMV model is illustrated in Fig. 1. It proposes a three-phase development of suicidal ideation, suicide attempts and suicide: the pre-motivational phase, the motivational phase, and the volitional phase. Although it was originally developed for suicidal behaviour, the IMV model is also applicable to self-harm [24]. When the core components of the model have been assessed in individuals who have self-harmed, it performs similarly to when it is applied to suicide attempts as the outcome, namely, higher levels of defeat and entrapment are associated with self-harm [24, 27]. Although not everyone who self-harms experiences suicidal ideation (i.e., having thoughts of death), many do and others experience thoughts of harming themselves without the intent to end one’s life (self-harm ideation). In addition, people who self-harm often report engaging in both non-suicidal self-injury and suicide attempts [25]. However, we acknowledge that the IMV model was not developed for NSSI specifically so, although we explore the pathway from suicidal ideation to NSSI in this study, this is not directly posited in the IMV model.

The first phase, the *pre-motivational phase*, contains background and triggering factors, including biological, genetic or cognitive vulnerability factors, and individual characteristics that increase the likelihood for suicide. The pre-motivational factors affect the risk of self-harm, suicide attempts and suicide through their influence on factors contained within the two next phases: the motivational and volitional phases.

The second phase, the *motivational phase*, can be decomposed into three elements: feelings of defeat and/or humiliation, feelings of entrapment and the development of suicidal ideation (or thoughts of self-harm). Defeat and entrapment are characterised by feelings of being brought down and unable to escape and are the key substrates associated with suicidal ideation. According to the model, existing pre-motivational factors can increase the risk of experiencing feelings of defeat/humiliation which can lead to feelings of entrapment and ultimately lead to the development of suicidal thoughts. In the IMV model, defeat and entrapment are prerequisites for the development of suicidal ideation, and therefore they are the backbone of the model. Moderating factors are important in understanding the transitions from defeat/humiliation to entrapment and from entrapment to suicidal ideation. The factors that influence the pathway from defeat to entrapment are the Threat to self-moderators (TSM) which include social problem solving, coping, memory biases and ruminative processes. The motivational moderators (MM) govern the transition from entrapment to suicidal ideation and include factors such as resilience, social support, goals, attitudes, burdensomeness, future thoughts, and thwarted belongingness. Each of these moderators might decrease or increase the risk of developing suicidal ideation.

The third and final phase, the *volitional phase*, describes the transition from suicidal thoughts to behaviour, i.e. this is the phase in which individuals may act on their thoughts. Volitional moderators (VM) are thought to either strengthen or weaken this transition, influencing whether an individual ultimately acts on their suicidal thoughts or not. The eight VMs include access to means, planning the suicidal act, exposure to suicide, impulsivity, physical pain sensitivity, fearlessness about death, imagery about dying or death and past suicidal behaviour [22].

Although the evidence is growing for the IMV model [34], to date, there have been limited attempts to explore its utility within adolescent population. In this survey we aimed to apply the IMV model to a sample of Danish youth aged 15–19 years.

Specifically, we explored:

i) whether the central tenets of the IMV model apply to a young Danish population in relation to intentional self-harm, including non-suicidal self-injury and suicide attempts as outcome measures, specifically the defeat – entrapment – ideation – behavior pathway.
ii) whether a selection of moderators from across the three phases of the IMV model are significant predictors of intentional self-harm, including non-suicidal self-injury and suicide attempts as outcome measures.

## Method

### Design and participants

A cross-sectional self-report survey design was employed. Every school in Denmark (around 2,400 schools) was invited through e-mail to participate in the study. If a school agreed to participate, parents were informed, and their consent was sought for their child to participate in the study. An electronic questionnaire was sent to schools who agreed to participate, and each respondent filled out the survey online.

### Measures

The survey consisted of a broad range of psychometric instruments which were translated into Danish following best practice guidelines, [2]. Before distribution, the questionnaire was piloted with students in the younger age group (14–15 years old) to assess its quality and identify possible sources of error.

Participants completed the following measures, all of which—except depression—are part of the IMV model.

**Defeat.** To measure defeat in the past 2 months, we used the 4 items related to defeat from the Short Defeat and Entrapment Scale (SDES). Responses are given on a 5-point scale ranging from 0 *“Not at all (completely disagree)”* to 4 “*Extremely like me (completely agree).* [10]

**Entrapment.** We used the 4 items from the Entrapment Short-Form Scale to measure internal and external entrapment in the past 2 months. The responses are made on a 5-point scale ranging from 0 *“Not at all like me (completely disagree)”* to 4 “*Extremely like me (completely agree)* [6].

**Rumination.** The Rumination Response Scale consists of 22 items. We used the 10 rumination items from the scale to measure current rumination when a person feels sad or depressed. Responses are made on a 4-point scale ranging from 1 “*Almost never*” to 4 “*Almost always*” [35].

**Thwarted Belongingness and Perceived Burdensomeness**. These were assessed using the 15-item Interpersonal Needs Questionnaire. The scale includes items to assess belongingness and burdensomeness in the past 2 months. Responses are made on a 7-point scale ranging from 1 *“Not at all true for me (completely disagree)”* to 7 “*Very true for me (completely agree)* [38].

**Social support.** Social support was measured using 5 items from the 7 items ENRICHD social support instrument (ESSI) [17]. We excluded the 2 items related to being adult. The scale includes 5 items and responses are made on a 5-point scale ranging from 0 *“Not at all (completely disagree)”* to 5 “*Extremely like me (completely agree)* and measures the level of current social support.

**Resilience.** The 10-item version of Connor-Davidson Resilience Scale (CD-RISC) was used to measure resilience in the past 2 months. The responses are made on a 5-point scale ranging from 0 *“Not at all true for me (completely disagree)”* to 4 “*True nearly all the time (completely agree)* (Campbell-Sills & Stein, 2007).

**Pain Tolerance and Fearlessness about death.** The Acquired Capability With Rehearsal for Suicide Scale (ACWRSS) was used to measure current pain tolerance and fearlessness about death. The scale has 7 items and responses are made on a 8-point scale ranging from 0 *“Not at all (completely disagree)”* to 8 “*Very strongly (completely agree)”* [9].

**Exposure to intentional self-harm.**

Exposure was assessed using two items on intentional self-harm among family members and close friends: “Has anyone among your family attempted suicide, deliberately harmed or killed themselves?” and “Has anyone among your close friends attempted suicide or deliberately harmed or killed themselves?” Response options were: 0 = No, 1 = Yes, within the last 12 months, and 2 = Yes, more than 12 months ago. These items have previously been used in IMV-model research to measure exposure to intentional self-harm [24]. For the present study, responses were dichotomized into ever exposed (categories 1 and 2) versus unexposed (category 0), regardless of type or timing of exposure.

**Impulsivity.** Impulsivity was assessed using an abridged version of the Barratt Impulsiveness Scale (BIS-11). We specifically used 5 items about level of Motor Impulsiveness, at current time. Responses are made on a 4-point scale ranging from 1 “*Rarely/Never”* to 4 “*Almost Always/Always”* [1, 26].

**Mental imagery.** Four items regarding mental images about own death were drawn from the paper by Holmes et al., [12]. The items include mental images about planning and acting out suicidal behavior, but also mental images of the world after own death by suicide, when the person feels sad or stressed. We used 4 items with responses ranged from 0 to 5. 0 = Never, 1 = Few times, 2 = Sometimes, 3 = Mostly and 5 = Always.

**Intentional self-harm.** These were assessed with 3 items (suicidal ideation, nonsuicidal self-injury and suicide attempts) drawn from Self-Injurious Thoughts and Behaviours Interview (SITBI) [21]. Participants were asked about the lifetime presence of suicide ideation, non-suicidal self-injury (NSSI) and suicide attempt. The items were slightly adapted to better suit the target group, the Danish language, and WHO recommendations for neutral and non‑stigmatizing suicide terminology. Additional questions were about behaviours in the past year, and past month, as well as the age of onset of each behaviour. Responses are made on 6-point scale ranging from “*Never*” to “*Five or more times*”. Consistent with the National Institute of Health and Care Excellence (NICE) guidelines, we have adopted the following definition for intentional self-harm: “intentional self-poisoning or injury, irrespective of the apparent purpose.” [20] As a result, intentional self-harm was operationalised as having ever engaged in either non-suicidal self-injury (NSSI) or a suicide attempt.

**Depression.** Depression Anxiety Stress Scale, The Depression subscale has 7 items and was used to measure depression in the past 2 months. Responses are made on a 4-point scale ranging from 0 “*Did not apply to me at all (completely disagree)*” to 3 “*Applied to me very much or most of the time (completely agree)”* [16]. Depression is not original included in the IMV-model, but we included as an exploratory addition.

### Statistical analysis

Descriptive analysis was used to analyse the demographic and psychosocial characteristics of the study population. Minimum, maximum, mean, and standard deviation were reported for all the instruments. Internal consistency of the measures was calculated using Cronbach’s alphas. Spearman’s or Pearson’s correlation between psychometric instruments and other factors were calculated.

#### Model fit of the defeat-entrapment-ideation-intentional self-harm or NSSI graph

We used pathway analysis for testing the fit of the IMV-model, which is our main analysis. We modelled the central tenets of the IMV model by excluding non-significant pathways from the full graph which included all possible forward pathways between the four factors. Age and sex were controlled for. The 3 pathways forming the central tenets (defeat, entrapment, ideation and outcome (Intentional Self-Harm or NSSI) were mandatory pathways. Two separate models were tested with Intentional Self-Harm (i.e., a composite of NSSI or suicide attempts) and NSSI as the primary outcome. The model with the best fit, including only significant pathways, was chosen. Statistics on model fit, standardized path coefficients and their significance level were reported, as recommended by [29]. The standardized coefficients were used to compare the relative magnitude of effects across variables. We used the package lavaan in R (4.3.2), to estimate pathway effects and calculating the model fit statistics. The BFGS optimization method, with DWLS estimates, which reports robust standard errors, was used.

#### Testing the effects of moderators on pathways

A structural hierarchical approach was used to analyse the effects of moderators on pathways. Effects were estimated in two different settings, in a crude setting and adjusted setting including confounders (age and sex). For pathways with a continuous exposure and a continuous outcome a linear regression was used (lm in R) and for pathways with binary or continuous exposure and binary outcome a logistic regression model was used (glm in R). Moderating effects were reported as both estimates and illustrations. For illustration, two curves were drawn between exposure and outcome covering that specific path of interest, for two different levels of the moderator (mean plus and minus one standard deviation). The Hayes process macro for SAS (9.4) was used for analysing moderators.

## Results

### Descriptive analysis

A total of 1,637 students from 11 schools participated in the study. Those who were older than 19 years old (*n* = 16) and those who had missing data in one or more measures (*n* = 40), were excluded. The final study population comprised of 1,581 individuals, of which 458 (29.0%) were from elementary school (aged 13 to 17 years), 380 (24.0%) from private primary school (aged 13 to 17 years), 717 (45,4%) from high school (aged 15–19 years) and 26 (1,6%) from special schools (aged 13 to 16 years). The dataset only had two response options regarding gender where 836 (52.9%) identified as girls and 745 as boys (47.1%). The average age was 15.8 years, with a standard deviation at 1.66 years.

30.1% reported that they had serious thoughts about suicide (36.4% of the girls, 23.1% of the boys, 19.0% with in last year, 7.9% within last month), 29.2% reported to have at least one episode of NSSI (40.3% of the girls, 16.6% of the boys, 17.7% within last year, 7.2% within last month) and 7.7% reported that they have attempted suicide (8.4% for the girls, 6.9% for the boys, 3.5% within last year, 1.4% within last month) at some stage in their lives. Combining individuals with NSSI or suicide attempts into a single category revealed that 30.6% had intentional self-harmed (40.9% of the girls, 19.1% of the boys, 21.2% within last year, 8.5% within last month). The lifetime prevalence of exposed to intentional self-harm was 49.3%.

The internal consistency between items in the psychometric instruments were generally high with Cronbach’s alpha values in the range from 0.60 to 0.90. Only fearlessness about death and pain tolerance are at a low and questionable level (see Table 1.). The correlations between the psychometric instruments and other factors included in this study can be found in Supplemental material 1 (SUP1).

**Table 1:** Internal realibility and descriptors for psychometric instruments

Psychometric instrument	Cronback’s alpha	Mean (sd)	Min-Max
Defeat	0.88	3.51 (3.69)	0–16
Entrapment	0.90	3.14 (3.96)	0–16
Depression	0.90	11.02 (4.40)	7–28
Rumination	0.89	18.18 (6.32)	10–40
Thwarted belongingness	0.85	17.07 (8.75)	7–49
Perceived burdensomeness	0.88	17.64 (8.47)	8–55
Social support	0.91	20.97 (4.14)	5–25
Resilience	0.90	24.74 (8.11)	0–40
Impulsivity	0.77	10.63 (3.01)	5–20
Fearlessness about death	0.60	16.19 (7.22)	5–40
Pain tolerance	0.67	7.70 (4.25)	2–16
Mental images	-	6.25 (2.85)	4–20

### Testing the central tenets of the IMV model

#### Pathway analysis with intentional self-harm as outcome

The model with Intentional Self-Harm as the outcome is seen in Fig. 2.a. It includes the mandatory paths along with additional paths from defeat to ideation and defeat to Intentional Self-Harm. The model fitting statistics are very good. The Adjusted Goodness of Fit is > 0.999 (GFIand the Normed Fit Index (NFI) is 0.996, exceeding the 0.95 threshold typically acceptable for small samples. The Comparative Fit Index (CFI) is a revised form of NFI and should be above 0.95, and it is 0.996. The Root Mean Square Error of Approximation (RMSEA), a parsimony-adjusted index, is 0.022, indicating a good fit, as values below 0.05 are considered ideal.

The standardized path coefficients are statistically significant and positive, ranging from 0.07 to 0.86 with highest values observed in the path from defeat to entrapment. The lowest values were found for defeat to Intentional Self-Harm and defeat to ideation, which is expected, as these are not hypothesized as direct pathways in the IMV model. The paths from defeat to ideation and Intentional Self-Harm are residual measures of effects, after controlling for the mandatory pathways in the model. As a result, not all individuals follow the pathways defined by the central tenets of the theory. Not all confounders were significant in all the paths and the size and sign of the effects were not equal in all the paths.

**Fig. 2a:**
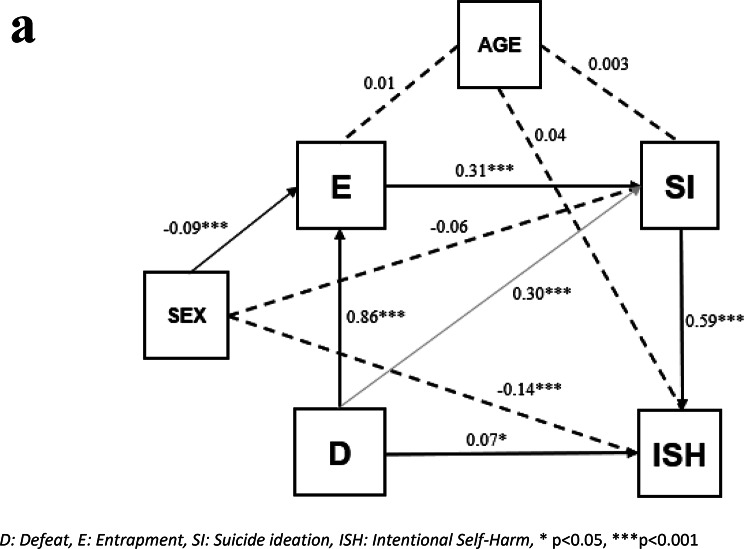
Pathway analysis of intentional self-harm. D: Defeat, E: Entrapment, SI: Suicide ideation, ISH: Intentional Self-Harm, * *p* < 0.05, ***p < 0.001

#### Pathway analysis with NSSI as outcome

The model with NSSI as the outcome can be seen in Fig. 2.b. It includes the mandatory paths but also the path from defeat to ideation and from entrapment to NSSI. Model fitting statistics are very good. (GFI > 0.999, NFI > 0.999, CFI > 0.999, RMSEA = 0.0005).

**Fig. 2b:**
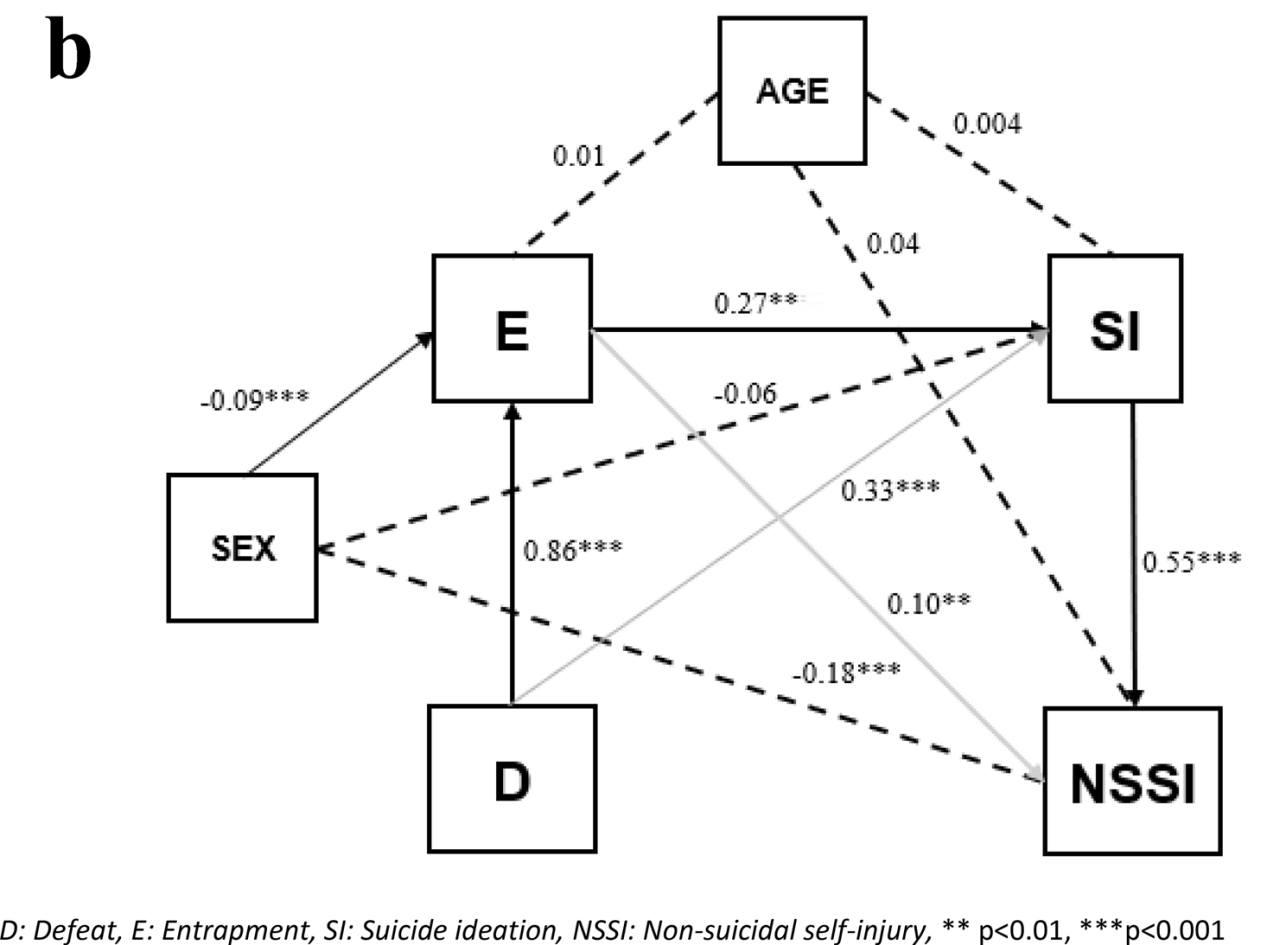
Pathway analysis of NSSI. D: Defeat, E: Entrapment, SI: Suicide ideation, NSSI: Non-suicidal self-injury, ** *p* < 0.01, ***p < 0.001

The standardized path coefficients are statistically significant, positive and in the range of 0.10 to 0.86 with highest values in the path from defeat to entrapment. The lowest value was found for entrapment to NSSI, which is expected, as this is not hypothesised as a direct pathway within the IMV model. The path from defeat to ideation is also not a pathway within the IMV model. The paths from defeat to ideation and entrapment to NSSI are residual measures of effects, after controlling for the mandatory pathways in the model. Not all confounders were significant in all the paths, and the size and sign of the effects were not equal in all the paths.

The pathway analysis with suicide attempts is reported in Supplemental material 2 (SUP2), showing similar results as for NSSI but a larger statistically significant path coefficient from suicidal ideation to suicide attempts (0.74).

#### Effects of moderators on pathways

The complete analysis of moderators on the pathways can be found in Supplemental material 3 (SUP3), reporting only the statistically significant moderators.

##### From defeat to entrapment

Rumination:

In both the crude and adjusted analysis rumination was a significant moderator of the relationship between defeat and entrapment. Higher levels of rumination strengthened the relationship between defeat and entrapment. The confounders only modestly reduced the moderating effect of rumination. R-Squared values were very high (crude model R^2^ = 0.79, adj. model R^2^ = 0.80).

Depression:

In both the crude and adjusted analysis depression was a significant moderator of the relationship from defeat to entrapment. Higher levels of depression strengthened the relationship between defeat and entrapment. The confounders only modestly reduced the moderating effect of depression. R-Squared values were very high (crude model R^2^ = 0.79, adj. model R^2^ = 0.80).

##### From entrapment to suicidal ideation

Depression:

Depression was a strong moderator in both the crude and adjusted analysis, with depression strengthening the effect from entrapment on the likelihood for suicidal ideation significantly. The confounders only modestly reduced the moderating effect of depression. R-Squared values were low in both models (crude model R^2^ = 0.30, adj. model R^2^ = 0.30).

Perceived burdensomeness:

The moderating effect of perceived burdensomeness on the pathway from entrapment to suicidal ideation was statistically significant in both the crude and adjusted analysis. High levels of perceived burdensomeness strengthened the effect of entrapment on the likelihood of suicidal ideation significantly. The effect was highest when levels of entrapment were lowest. R-squared values were low in both models (crude model R^2^ = 0.28, adj. model R^2^ = 0.28).

Non-significant results.

Lack of social support, high feeling of thwarted belongingness, or low degree of resilience were raising the effect on suicidal ideation from entrapment, but with statistically non-significant effects.

##### From suicidal ideation to Intentional Self-Harm

Mental imagery:

There was clear evidence of mental imagery moderating the pathway from suicidal ideation to Intentional Self-Harm. The effect was statistically significant in both the crude and adjusted model. In both cases the R-Squared values were low, and sex had some effect on the process (crude model R^2^ = 0.33, adj. model R^2^ = 0.35).

Non-significant results.

High fearlessness about death, high pain tolerance, high impulsivity or being exposed to intentional self-harm were raising the effect from suicidal ideation on the risk for intentional self-harm, but with statistically non-significant effects.

##### From suicidal ideation to NSSI

Mental imagery:

There was clear evidence of mental imagery moderating the pathway from suicidal ideation to NSSI. The effect was statistically significant in both the crude and adjusted model. In both cases the R-Squared values were low, and sex had some effect on the process (crude model R^2^ = 0.30, adj. model R^2^ = 0.33).

Non-significant results.

High fearlessness about death, high pain tolerance, high impulsivity or being exposed to intentional self-harm were raising the effect from suicidal ideation on the risk for NSSI, but with statistically non-significant effects.

##### From suicidal ideation to suicide attempt

Almost all the volitional moderators were associated with strengthened relationships between suicidal ideation and suicide attempts, but with statistically non-significant effects. The risk of suicide attempts was consistently lower than that of intentional self-harm and non-suicidal self-injury across all combinations of suicidal ideation and levels of the moderating factor.

## Discussion

In this study we tested the central tenets of the IMV model in a young Danish population for the first time. We found a strong fit for the data on models for intentional self-harm (i.e., suicide attempts and NSSI) and NSSI as outcomes. Although statistically significant, the lowest effect was found for the entrapment to suicidal ideation pathways. However, this may have been explained, in part, by the direct effect of defeat on suicidal ideation and the shared correlation between defeat and entrapment. We also found that the following moderators: rumination, depression, thwarted belongingness, perceived burdensomeness, and mental imagery were significant moderators within the motivational/volitional phases of the model, as predicted. The rest of the moderators included in this study were statistically non-significant.

### Comparison with other ideation-to-action models

The IMV model, similar to the interpersonal theory of suicide (IPTS) [39], the three-step theory (3ST) [13] and the fluid vulnerability theory (FVT) [30] helps clinicians and stakeholders to better understand the suicidal process. All four models consider the process from background factors to suicidal ideation and from suicidal ideation to suicidal behaviour, as two distinct processes. They also assume that the ability to act on suicidal thoughts is dynamic and unstable over time and place. The pathway to suicidal ideation is more related to pain and hopelessness and the process from ideation to action is more related to the capability to act. Arguably, the IMV model is the most complex model which was built upon IPTS and includes components from the 3ST model. The FVT model also overlaps considerably with the three other models [14].

### The central tenets of the IMV model

We analysed the central tenets of the IMV model with three different outcomes: Intentional Self-Harm, NSSI or suicide attempts. We ended up with almost identical models consistent with the IMV model, however, the models also included a direct effect of defeat on suicidal ideation. We were also surprised that the mediating effect of entrapment on the relationship between defeat and the ideation was not stronger. In our sample, therefore, the pathway from defeat to ideation was only partly mediated by entrapment, as we had a direct effect and a mediating effect. The lowered standardised path coefficients from entrapment to ideation in the models can be explained by the model which also includes the direct effect from defeat to ideation. Some other studies of young people also found low or moderate effects size from entrapment to suicidal ideation, as in the IMV model test in a young Chinese population [15] and in the study of college students by Moscardini et al., [18]. A meta-analysis of Pearson’s correlation between defeat, entrapment and suicidality found defeat and entrapment to be clinically important in suicidality, with correlation effect sizes around 0.60, for both defeat and entrapment on suicidality [33]. In our models, the path from suicidal ideation to suicide attempts (β = 0.74) was stronger than the path to NSSI (β = 0.55), even though the correlation between suicidal ideation and NSSI was higher than that between suicidal ideation and suicide attempts. This pattern suggests that, although suicidal ideation and NSSI are closely related, the IMV model provides a better explanatory fit for suicide attempts than for NSSI when controlling for other factors. These findings should be interpreted in light of the low base rate of suicide attempts, which reduces the precision of the coefficient for the ideation-to-attempt pathway. The strong association between ideation and NSSI indicates considerable overlap between these behaviors, which cannot be fully explained by defeat, entrapment, or other factors in the IMV model. Psychologically, this aligns with previous research suggesting that individuals who self-injure may experience suicidal thoughts intermittently, even when their primary intent is non-suicidal [40]. These findings highlight the importance of assessing suicidal ideation in individuals presenting with NSSI, as its presence may signal elevated risk for progression to suicidal attempts.

Only a limited number of studies have examined the central tenets of the Integrated Motivational–Volitional (IMV) model within a single analysis, as we have done in the present study [34]. Despite differences in sample characteristics, their findings align closely with ours. All three studies use cross‑sectional designs, but different instruments are employed to assess the four IMV constructs. Defeat and entrapment are measured as present‑state constructs, whereas suicidal ideation and self‑harm/suicide attempts vary in timeframe. Across all studies, the weakest association among the core IMV pathways was between entrapment and suicidal ideation. Nevertheless, the indirect pathway from defeat to suicide attempts - mediated by entrapment and suicidal ideation - was consistently supported. The existing evidence spans diverse populations: a youth sample from China [15], adult university students [7], and adult prisoners [32], suggesting that the IMV model’s central mechanisms are robust across different demographic and contextual settings.

### Moderators

#### Threats to self and motivational moderators

Rumination and depression were significant moderators in the path from defeat to entrapment. This fits with previous studies that found rumination to be correlated with and predictive of suicidal behaviour among depressed individuals [4] and among individuals with low sleep quality [11]. Another study using non-parametric bootstrapping procedures found that subtypes of rumination: brooding, but not reflection, moderates the relationship between defeat and entrapment [36]. The same moderating effect was not found in a model test of the IMV model using young Chinese adolescents [15]. We found strong evidence that rumination is moderating the relationship between defeat and entrapment.

As in other studies of adolescents we found thwarted belongingness and perceived burdensomeness to be important motivational moderators [15]. These two factors are central in the IPTS-model [39]. A meta-analysis found that both thwarted belongingness and perceived burdensomeness were more strongly related to suicidal ideation and suicide risk than suicide attempts [5]. We found a non-significant trend for social support, which may have been affected by low statistical power. Social isolation is a strong predictor for suicidal ideation, attempts, and lethal suicidal behaviour across lifespan [39] and a large review concludes that for young age groups a fight against social isolation looks like a major lead for suicide prevention [19]. Often social support is handled and analysed as a direct risk factor for suicidal behaviour, but not in the IPTS or IMV model, where it is included as a direct risk factor or a moderator for suicidal ideation [14]. We found strong negative correlation between levels of social support and thwarted belongingness, as thwarted belongingness might lead to low level of social support.

We found only a moderate and non-significant effect from resilience as a moderator on the path from entrapment to ideation. Others have found a more clear pattern where resilience was a significant moderator on the path [15] and another study found significant higher level of resilience among controls than individuals with suicidal ideation/suicide attempt. The association became non-significant after adjusting for other moderators, including some volitional moderators [41]. Future research could usefully explore whether resilience is as important in an adolescent context compared to other age groups.

#### Volitional moderators

All of the ideation-to-action models have a large potential to inform life-saving interventions, especially in terms of reducing individuals’ capability for acting on suicidal ideation. The IMV model is not only focusing on individuals’ capability for acting on suicidal ideation, but also on other circumstances related to volitional moderators. In total, five volitional factors from the IMV model were included in the different models. All the moderators were clinically relevant, but not all were statistically significant, probably due to low statistical power, as suicidal ideation, NSSI, and suicide attempts were relatively rare events. Suicide attempts were reported by less than 8% of the sample, so these analyses need to be interpreted with extreme caution. Other studies found similar results, highlighting the importance of the volitional factors [15, 34, 41]. The IMV model was originally developed for suicide attempts, thus the volitional moderators were used to distinguish between those with only suicidal ideation, and those acting on the ideation. However, studies show that the volitional moderators are also relevant moderators in the pathway from ideation to NSSI [22, 24]. This study strengthens that hypothesis.

### Depression as a moderator

Depression is not originally included in the IMV model, either as a confounder or a moderator. Major depressive disorder and depressive symptoms are among the strongest predictors of suicidal behavior [28]. We included an exploratory analysis of depression as both a threats-to-self- and a motivational moderator, to assess its relevance in the IMV model. In both cases depression significantly moderates the pathways, such that higher levels of depression strengthen the correlations between defeat and entrapment, as well as between entrapment and suicidal ideation. Feelings of defeat and entrapment are often elevated in individuals with depression and Intentional Self-Harm, and have been theoretically linked to the development and maintenance of psychiatric disorders, indeed depression itself might also lead to feelings of defeat and entrapment [33]. We found it to be among the strongest moderators in this study and would recommend researchers and clinicians to explore this further.

### Clinical implications

The IMV model is one model, among others, to explain the suicidal process from background factors to suicidal behavior. The model is part of the ideation-to-action group and is trying to differentiate between those who do not act and those who act on their suicidal ideation. The process towards intentional self-harm can be strengthened or weakened by threat-to-self or motivational moderators, which are therefore important in the early prevention of intentional self-harm. In this study, we found that high levels of depression, rumination, and perceived burdensomeness are significantly associated with the pathways leading towards Intentional Self-Harm. The evidence of the moderators is less empirically evidenced and needs to be explored further [34]. Much time and resources are spent every day on clinical suicide risk assessment using predictive instruments, but probably with low clinical value as this requires an adequate positive predictive value (PPV), which is not the case for suicide risk [3, 31]. This study had difficulty documenting the statistical significance of the volitional moderators, as it requires a very large sample size to identify significant moderating effects between the two rare factors: suicidal ideation and intentional self-harm. We recommend testing whether there is potential for better/more accurate psychosocial assessment by including all volitional moderators in the comprehensive and compassionate assessment of those at risk of suicide. Incorporating the IMV model allows clinicians to focus on specific psychological factors like rumination or perceived burdensomeness, offering a more personalized approach. This dynamic assessment can help identify individual pathways, enabling more targeted interventions for those at risk of suicidal behavior.

### Strengths and limitations

This study has several strengths, including a detailed assessment of a large sample from across Denmark. However, because the study design was cross-sectional, we cannot determine temporal relationships between the factors or draw conclusions about causality. There is also a risk of reverse causality, as suicidal ideation and self‑injurious behavior were assessed using lifetime measures, which may artificially inflate their associations with present‑state (or two‑month‑state) constructs such as defeat, entrapment, and other included measures. Alternative longitudinal study designs are needed to establish temporal precedence between factors within the context of the IMV model. Unfortunately, we did not have a measure of thoughts of NSSI. Future research should explore the thoughts of NSSI to NSSI replacement.”

Although the overall sample size was large, the number of cases involving suicide attempts was modest (7.7%). This low base rate and other rare exposures limit statistical power for detecting moderation effects, which require sufficient variability in both the outcome and the interacting variables. When both the exposure and the outcome are relatively rare, estimates become less precise, confidence intervals widen, and the risk of Type II error increases. Using lifetime measures was appropriate, as lifetime history is a robust risk marker that captures clinically meaningful variance and provided more stable estimates and greater statistical power than shorter recall periods. Consequently, while our findings provide initial evidence for the IMV model pathways, more highly powered studies - ideally with larger longitudinal samples or pooled data - are needed to robustly examine potential moderators and confirm true psychological processes rather than sampling variability.

Additionally, the sample is not representative of Danish youth, as participation in the study was voluntary and only a small number of the invited schools agreed to take part. Nevertheless, elementary schools, high schools, private primary schools, and special schools are included, covering most of the school types available to youth in Denmark. It was not possible to compare the socio-demographic characteristics of participants and non-participants, so we cannot determine whether the sample is biased toward specific socio-demographic groups. Furthermore, the translated psychometric instruments have not undergone additional psychometric validation in the Danish version, and we did only include two gender options (boy, girl).

## Conclusion

Findings from a population of young Danish individuals show patterns broadly consistent with the central tenets of the IMV model. The association between entrapment and suicidal ideation, while significant, emerged as the weakest of the central tenets. Evidence also suggests that the IMV framework may be relevant across both NSSI and suicide attempt contexts in this population. However, given the cross-sectional design, these results should be interpreted with caution, as temporal precedence and causal relationships cannot be inferred. Further longitudinal research is needed to confirm whether the IMV model reliably predicts the onset and repetition of suicidal behavior over time. Despite these limitations, the study underscores the potential value of assessing defeat, entrapment, and volitional moderators in clinical practice, as such factors may help identify individuals at elevated risk and inform targeted interventions.

## Supplementary Information

Below is the link to the electronic supplementary material.

Supplementary Material 1

## Data Availability

The data cannot be shared with third parties because no participant consent was obtained for such disclosure.
