# Supplementary material for: Testing the Integrated Motivational-Volitional Model of suicidal behavior in a young Danish population
Source: BMC Psychiatry. 2026 Apr 10;26:398. doi: 10.1186/s12888-026-08049-2 (PMC13185257; doi:10.1186/s12888-026-08049-2)
Supplement: Supplementary file 1 — Supplementary Material 1 [file 12888_2026_8049_MOESM1_ESM.docx]

Supplemental material 1 (SUP1). Spearman’s or Pearson’s correlation between psychometric instruments and other factors used in this study

|  | 1 | 2 | 3 | 4 | 5 | 6 | 7 | 8 | 9 | 10 | 11 | 12 | 13 | 14 | 15 | 16 | 17 | 18 | 19 |
| --- | --- | --- | --- | --- | --- | --- | --- | --- | --- | --- | --- | --- | --- | --- | --- | --- | --- | --- | --- |
| 1. Suicidal ideation (SI) |  |  |  |  |  |  |  |  |  |  |  |  |  |  |  |  |  |  |  |
| 2. Non-suicidal self-injury (NSSI) | 0.50*** |  |  |  |  |  |  |  |  |  |  |  |  |  |  |  |  |  |  |
| 3. Suicidal attempt (SA) | 0.42*** | 0.33*** |  |  |  |  |  |  |  |  |  |  |  |  |  |  |  |  |  |
| 4. Intentional self-harm (ISH) | 0.54*** | 0.97*** | 0.43*** |  |  |  |  |  |  |  |  |  |  |  |  |  |  |  |  |
| 5. Defeat | 0.48*** | 0.41*** | 0.26*** | 0.42*** |  |  |  |  |  |  |  |  |  |  |  |  |  |  |  |
| 6. Entrapment | 0.48*** | 0.44*** | 0.26*** | 0.45*** | 0.87*** |  |  |  |  |  |  |  |  |  |  |  |  |  |  |
| 7. Depression | 0.49*** | 0.42*** | 0.27*** | 0.43*** | 0.78*** | 0.79*** |  |  |  |  |  |  |  |  |  |  |  |  |  |
| 8. Rumination | 0.42*** | 0.43*** | 0.23*** | 0.44*** | 0.61*** | 0.66*** | 0.66*** |  |  |  |  |  |  |  |  |  |  |  |  |
| 9. Thwarted belongingness | 0.35*** | 0.28*** | 0.21*** | 0.29*** | 0.61*** | 0.60*** | 0.60*** | 0.45*** |  |  |  |  |  |  |  |  |  |  |  |
| 10. Perceived burdensomeness | 0.40*** | 0.31*** | 0.25*** | 0.33*** | 0.69*** | 0.69*** | 0.69*** | 0.49*** | 0.62*** |  |  |  |  |  |  |  |  |  |  |
| 11. Social support | -0.28*** | -0.19*** | -0.18*** | -0.21*** | -0.48*** | -0.46*** | -0.46*** | -0.30*** | -0.60*** | -0.49*** |  |  |  |  |  |  |  |  |  |
| 12. Resilience | -0.28*** | -0.28*** | -0.16*** | -0.28*** | -0.43*** | -0.40*** | -0.41*** | -0.35*** | -0.42*** | -0.38*** | 0.30*** |  |  |  |  |  |  |  |  |
| 13. Impulsivity | 0.17*** | 0.19*** | 0.16*** | 0.18*** | 0.15*** | 0.16*** | 0.21*** | 0.22*** | 0.04*** | 0.14*** | -0.10*** | 0.04 |  |  |  |  |  |  |  |
| 14. Exposure | 0.26*** | 0.28*** | 0.12*** | 0.27*** | 0.19*** | 0.21*** | 0.22*** | 0.30*** | 0.10*** | 0.16*** | -0.07** | -0.14*** | 0.13*** |  |  |  |  |  |  |
| 15. Fearlessness about death | 0.38*** | 0.23*** | 0.28*** | 0.25*** | 0.43*** | 0.43*** | 0.47*** | 0.29*** | 0.35*** | 0.48*** | -0.32*** | -0.22*** | 0.19*** | 0.11*** | |  |  |  |  |
| 16. Pain tolerance | 0.21*** | 0.19*** | 0.15*** | 0.21*** | 0.16*** | 0.15*** | 0.19*** | 0.19*** | 0.10*** | 0.14*** | -0.09** | 0.17*** | 0.18*** | 0.13*** | 0.17*** |  |  |  |  |
| 17. Mental images | 0.49*** | 0.42*** | 0.29*** | 0.44*** | 0.57*** | 0.59*** | 0.62*** | 0.56*** | 0.41*** | 0.56*** | -0.33*** | -0.31*** | 0.22*** | 0.29*** | 0.49*** | 0.23*** | |  |  |
| 18. Sex | -0.14*** | -0.26*** | -0.03 | -0.24*** | -0.20*** | -0.28*** | -0.23*** | -0.37*** | -0.09** | -0.13*** | -0.01 | 0.28*** | 0.07** | -0.30*** | 0.14*** | 0.06* | -0.22*** | |  |
| 19. Age | -0.03 | 0.02 | -0.02 | 0.02 | -0.07** | -0.03 | 0.01 | 0.03 | -0.06* | -0.11*** | 0.11** | 0.10*** | -0.02 | 0.13*** | -0.14*** | 0.002 | -0.02 | -0.02 |  |

p<0.05, **p<0.01, ***p<0.001

**Supplemental material 2 (SUP2). Pathway analysis with suicide attempt as outcome**

The model fit with suicide attempts as the outcome can be seen in figure sup 3. It includes the paths from defeat to entrapment, entrapment to ideation, and ideation to self-harm, but also the paths from defeat to ideation and entrapment to suicide attempts, which are not hypothesised as direct paths in the IMV model. Model fit statistics are very good and only marginally worse than the model with self-harm as outcome. The GFI is 0.999, CFI is 1.000 and NFI is 0.999 and RMSEA is 0.006. The data is fitting the model very well.

Figure sup 3. Pathway analysis of suicide attempts.


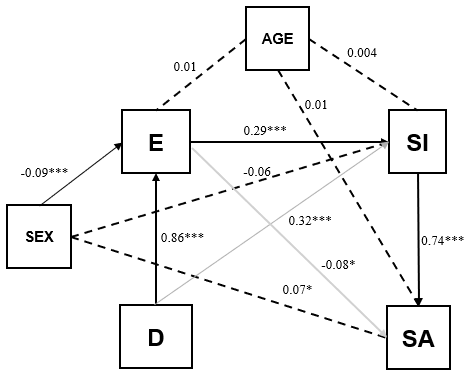


*D: Defeat, E: Entrapment, SI: Suicide ideation, SA: Suicide attempt*

* p<0.05, ***p<0.001

The standardized path coefficients were statistically significant and in the range of -0.08 to 0.86 with highest values in the paths from defeat to entrapment and suicidal ideation to suicide attempt. Consistent with the self-harm model, the lowest value was found for entrapment to suicide attempts. The negative path coefficients indicate some inverse relationship between entrapment and suicide attempts, after controlling for the direct effects of entrapment to ideation and ideation to suicide attempts. Not all confounders were significant in all the paths and the size and sign of the effects are not equal in all the paths (see figure sub 3 for more details).

**Supplemental material 3 (SUP3). Analysis of moderators**

**Moderators in path from defeat to entrapment**

| 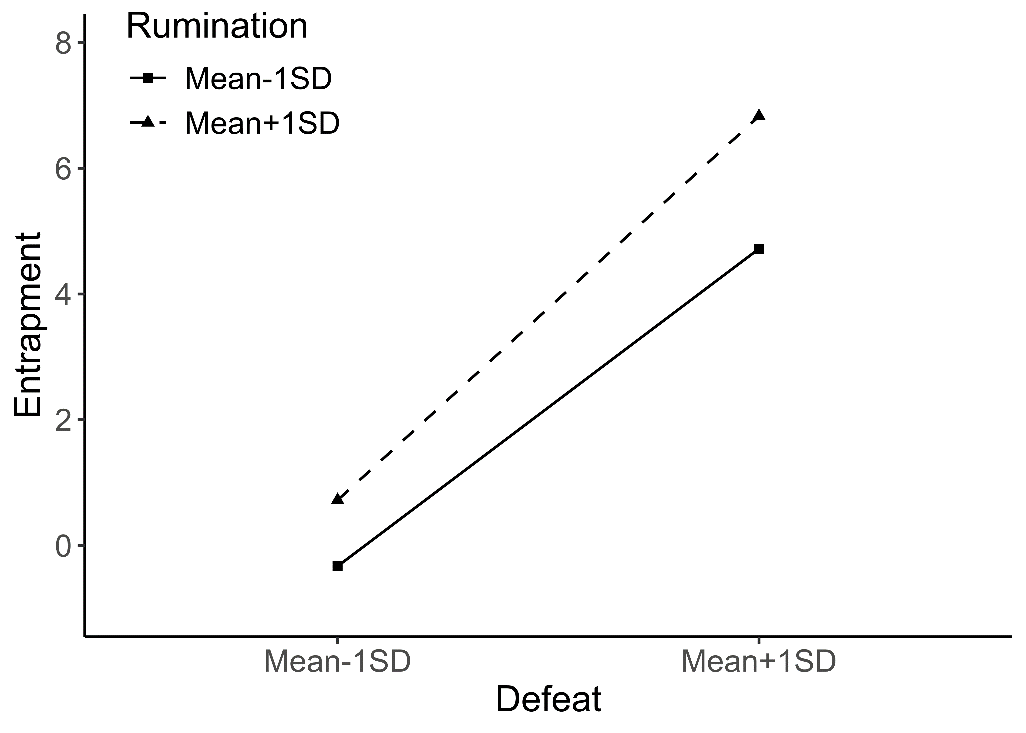 | 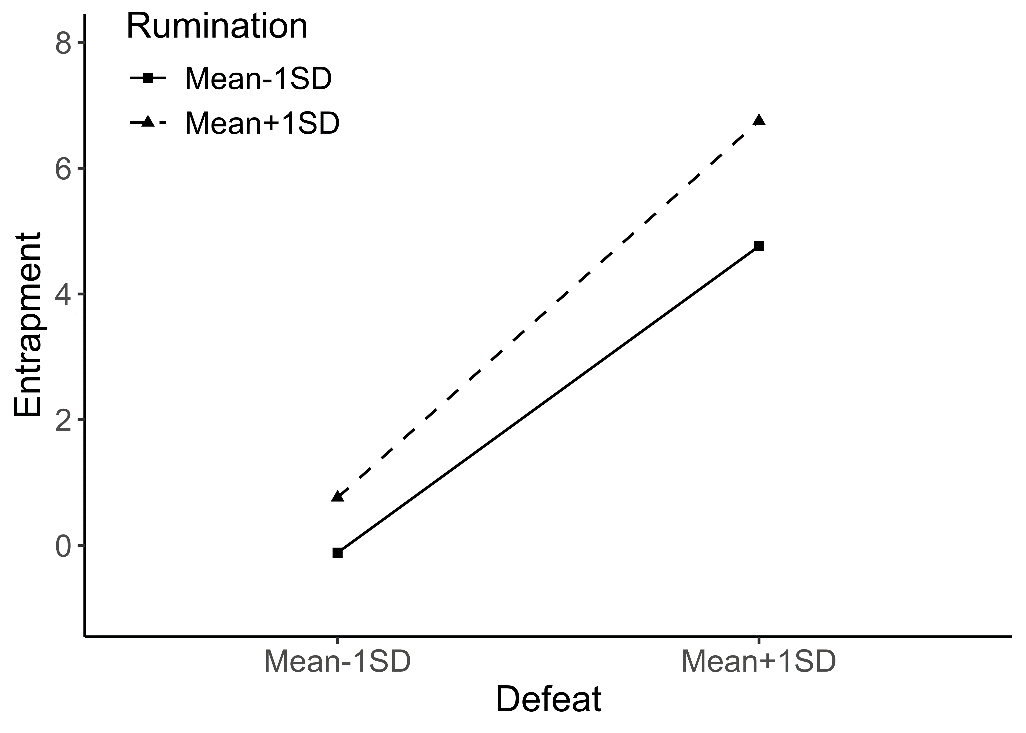 |
| --- | --- |
| \| **Name** \| **Estimate** \| **95% CI** \| \| --- \| --- \| --- \| \| Defeat \| 0.51*** \| 0.44 to 0.59 \| \| Rumination \| 0.14*** \| 0.10 to 0.17 \| \| Interaction \| 0.29*** \| 0.20 to 0.38 \|   R^2^=0.79, AIC=2012 | \| **Name** \| **Estimate** \| **95% CI** \| \| --- \| --- \| --- \| \| Defeat \| 0.50*** \| 0.42 to 0.57 \| \| Rumination \| 0.11*** \| 0.07 to 0.15 \| \| Interaction \| 0.32*** \| 0.23 to 0.41 \| \| Confounder: \|  \|  \| \| Sex \| -0.05*** \| -0.08 to -0.03 \| \| Age \| -0.01 \| -0.03 to 0.02 \|   R^2^=0.80, AIC=1997 |

* p<0.05, ** p<0.01, ***p<0.001

**Moderators in path from defeat to entrapment**

| 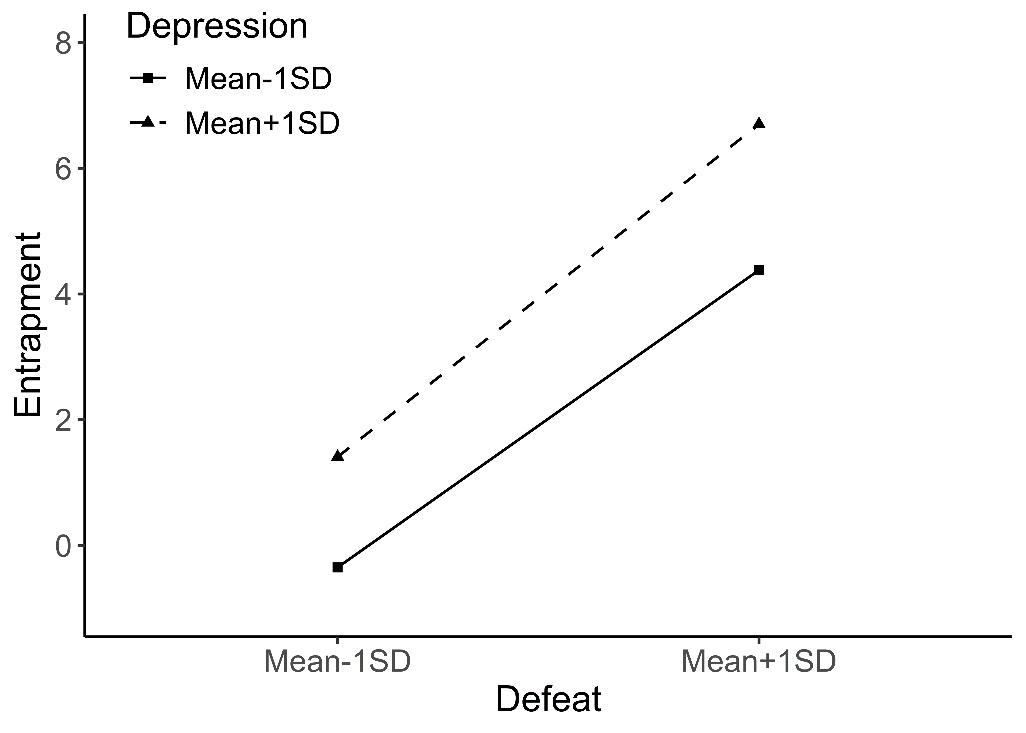 | 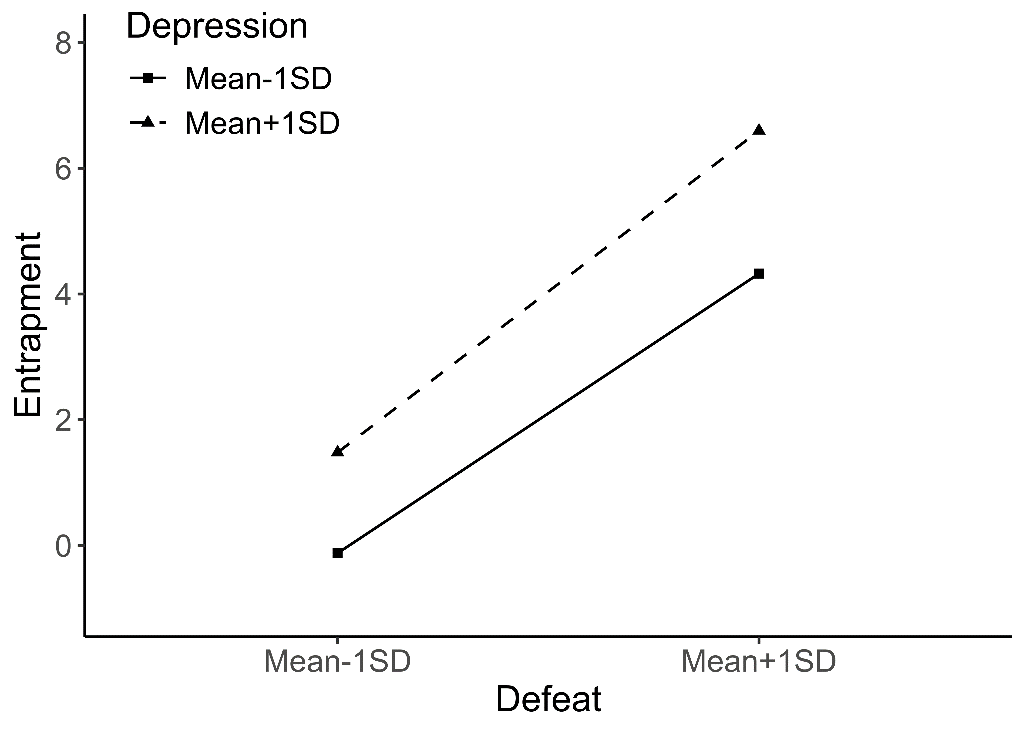 |
| --- | --- |
| \| **Name** \| **Estimate** \| **95% CI** \| \| --- \| --- \| --- \| \| Defeat \| 0.54*** \| 0.48 to 0.61 \| \| Depression \| 0.22*** \| 0.18 to 0.27 \| \| Interaction \| 0.16*** \| 0.08 to 0.25 \|   R^2^=0.79, AIC=2010 | \| **Name** \| **Estimate** \| **95% CI** \| \| --- \| --- \| --- \| \| Defeat \| 0.51*** \| 0.44 to 0.57 \| \| Depression \| 0.20*** \| 0.15 to 0.25 \| \| Interaction \| 0.20*** \| 0.12 to 0.29 \| \| Confounder: \|  \|  \| \| Sex \| -0.09*** \| -0.11 to -0.06 \| \| Age \| -0.01 \| -0.03 to 0.01 \|   R^2^=0.80, AIC=1959 |

* p<0.05, ** p<0.01, ***p<0.001

**Moderators in path from entrapment to suicide ideation**

| 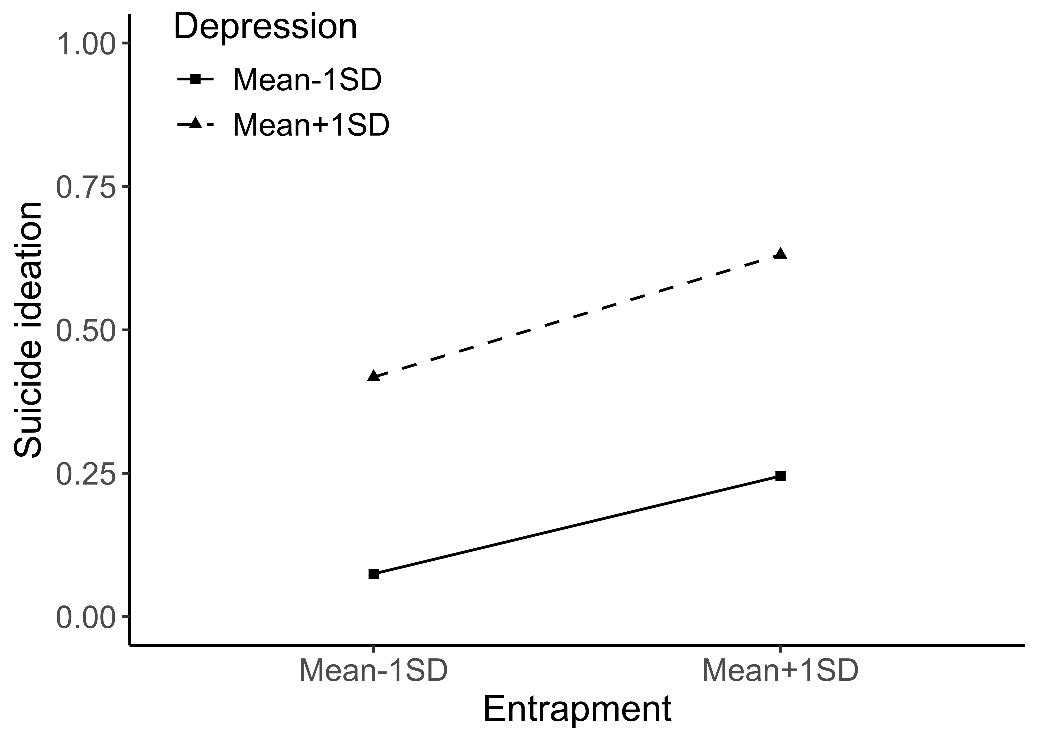 | 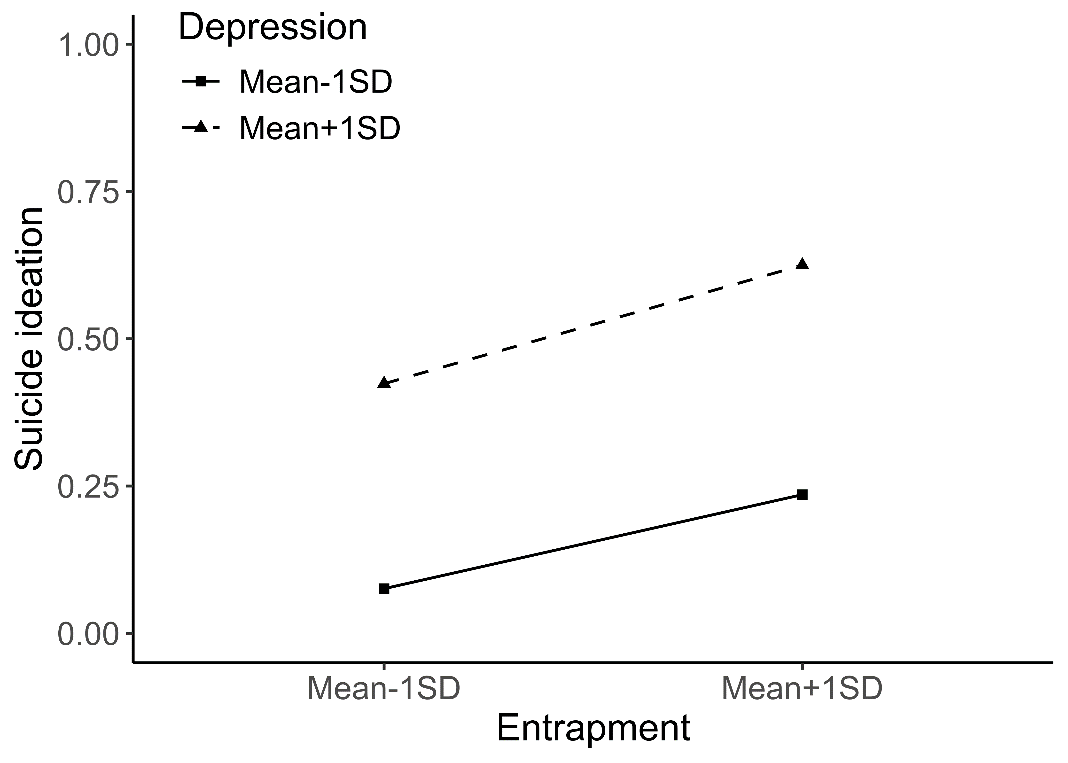 |
| --- | --- |
| \| **Name** \| **Estimate** \| **95% CI** \| \| --- \| --- \| --- \| \| Entrapment \| 0.99*** \| 0.54 to 1.42 \| \| Depression \| 1.09*** \| 0.84 to 1.35 \| \| Interaction \| -0.67* \| -1.23 to -0.05 \|   R^2^=0.30, AIC=1459 | \| **Name** \| **Estimate** \| **95% CI** \| \| --- \| --- \| --- \| \| Entrapment \| 0.95*** \| 0.50 to 1.38 \| \| Depression \| 1.10*** \| 0.84 to 1.36 \| \| Interaction \| -0.64* \| -1.21 to -0.02 \| \| **Confounder:** \|  \|  \| \| Sex \| -0.08 \| -0.22to 0.05 \| \| Age \| -0.06 \| -0.19 to 0.07 \|   R^2^=0.30, AIC=1461 |

* p<0.05, ** p<0.01, ***p<0.001

**Moderators in path from entrapment to suicide ideation**

| 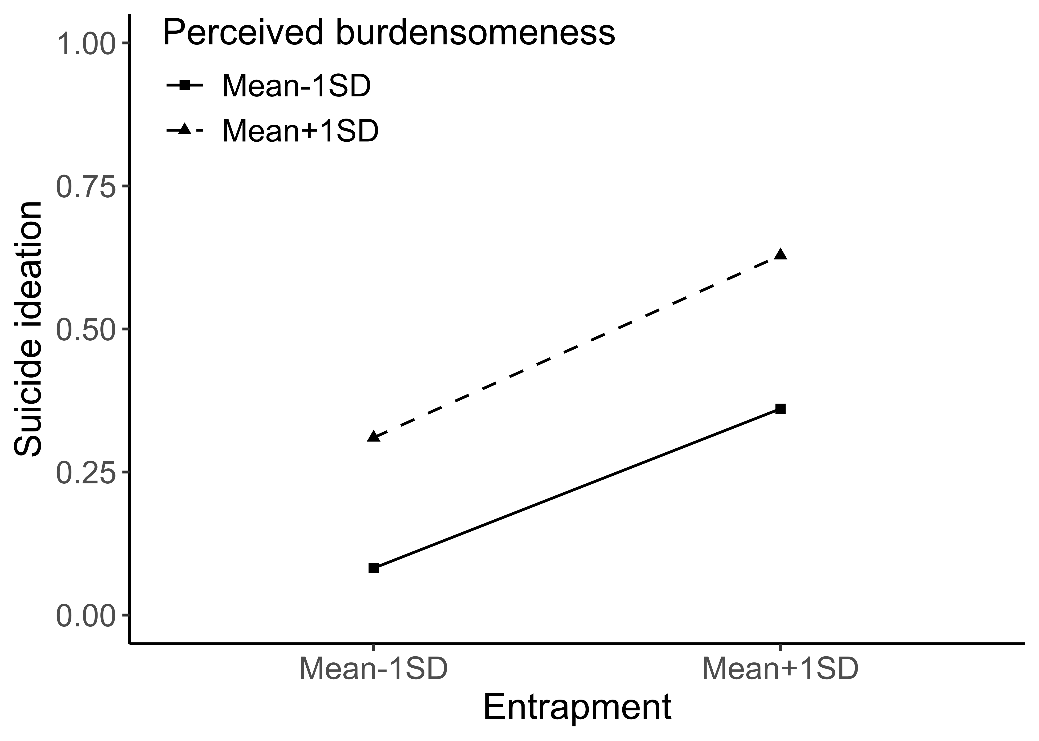 | 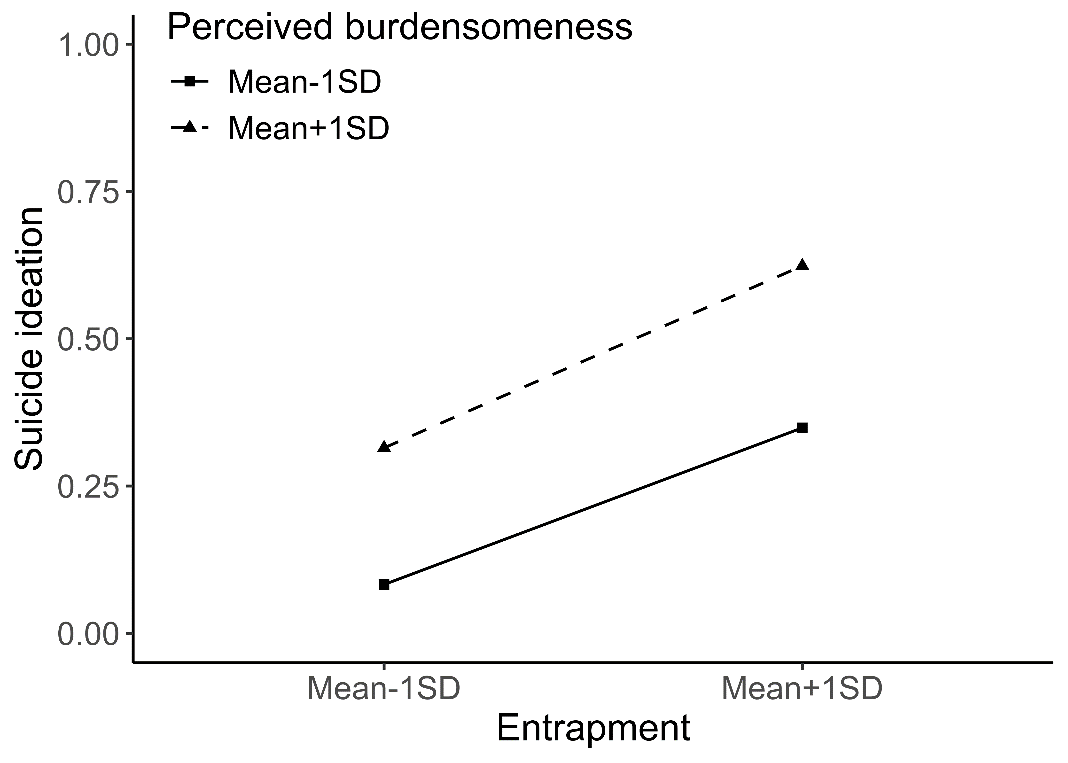 |
| --- | --- |
| \| **Name** \| **Estimate** \| **95% CI** \| \| --- \| --- \| --- \| \| Entrapment \| 1,18*** \| 0,82 to 1,54 \| \| Perceived Burdensomeness \| 0,81*** \| 0,56 to 1,06 \| \| Interaction \| -0,60* \| -1,10 to -0,05 \|   R^2^=0.28, AIC=1496 | \| **Name** \| **Estimate** \| **95% CI** \| \| --- \| --- \| --- \| \| Entrapment \| 1.14*** \| 0.77 to 1.50 \| \| Perceived Burdensomeness \| 0.81*** \| 0.56 to 1.07 \| \| Interaction \| -0.57* \| -1.07 to -0.02 \| \| **Confounder:** \|  \|  \| \| Sex \| -0.09 \| -0.22to 0.04 \| \| Age \| 0.05 \| -0.08 to 0.17 \|   AIC:0.28, R^2^=1497 |

* p<0.05, ** p<0.01, ***p<0.001

**Moderators in path from entrapment to suicide ideation**

| 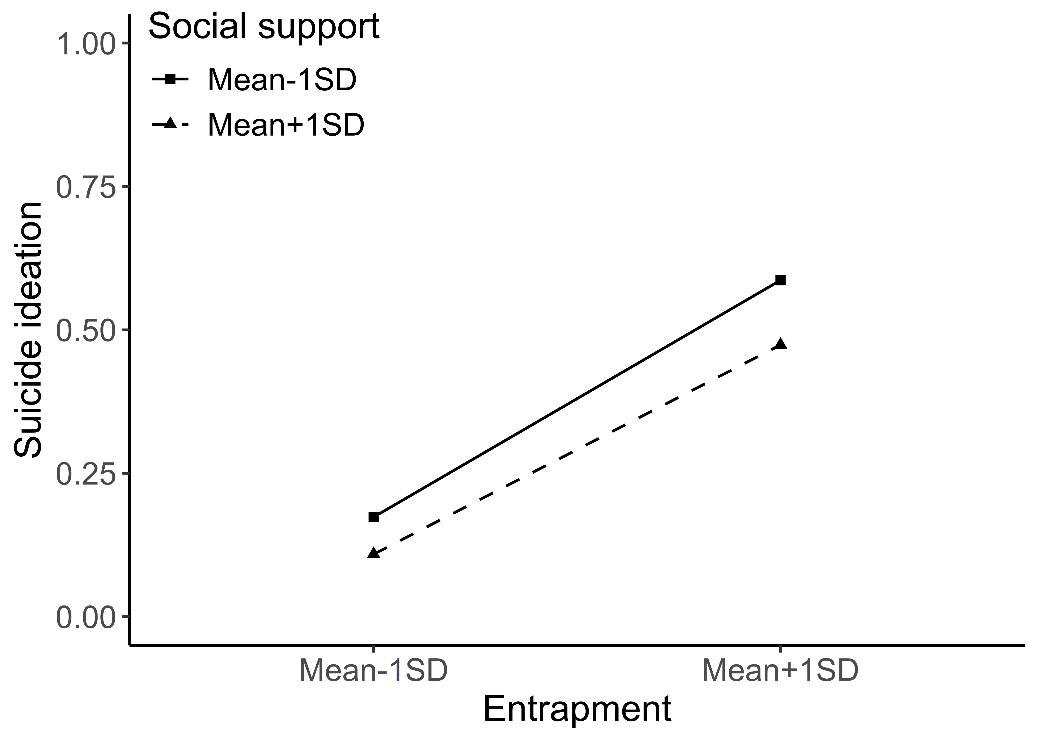 | 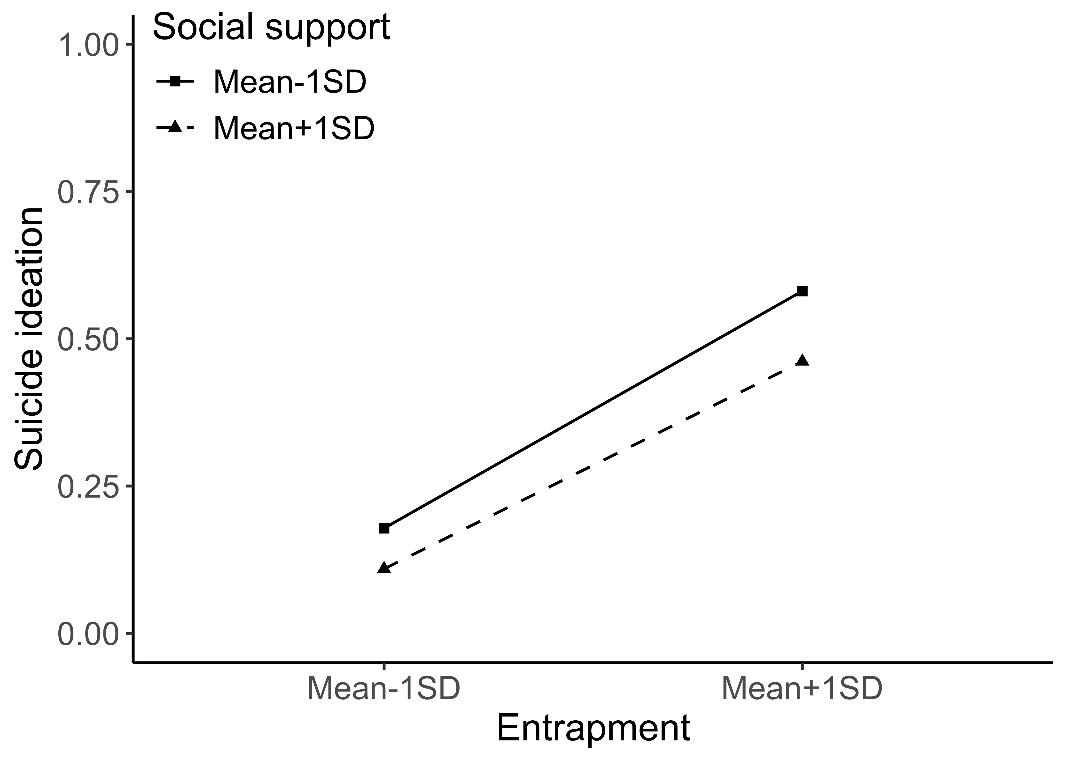 |
| --- | --- |
| \| **Name** \| **Estimate** \| **95% CI** \| \| --- \| --- \| --- \| \| Entrapment \| 0,96* \| 0,37 to 1,62 \| \| Social support \| -0,27* \| -0,46 to -0,08 \| \| Interaction \| 0,11 \| -0,47 to 0,65 \|   R^2^=0.26, AIC=1537 | \| **Name** \| **Estimate** \| **95% CI** \| \| --- \| --- \| --- \| \| Entrapment \| 0.93* \| 0.34 to 1.59 \| \| Social support \| -0.28* \| -0.47 to -0.09 \| \| Interaction \| 0.11 \| -0.47 to 0.65 \| \| **Confounder:** \|  \|  \| \| Sex \| -0.11 \| -0.24 to 0.02 \| \| Age \| 0.005 \| -0.12 to 0.13 \|   R^2^=0.26, AIC=1538 |

* p<0.05, ** p<0.01, ***p<0.001

**Moderators in path from entrapment to suicide ideation**

| 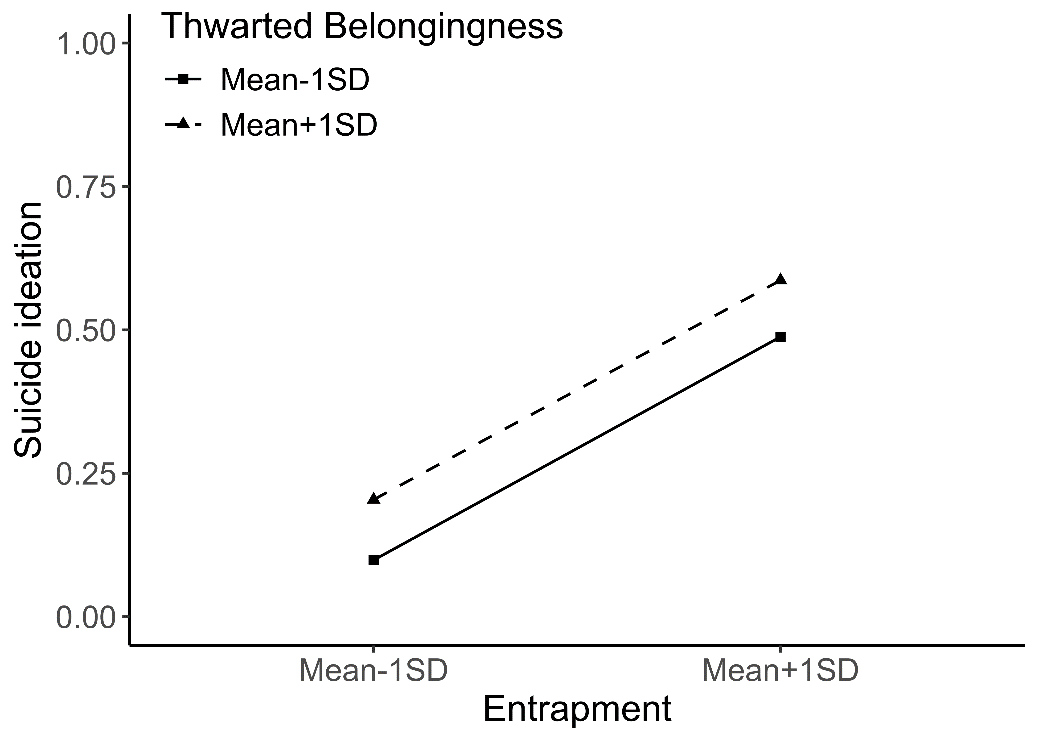 | 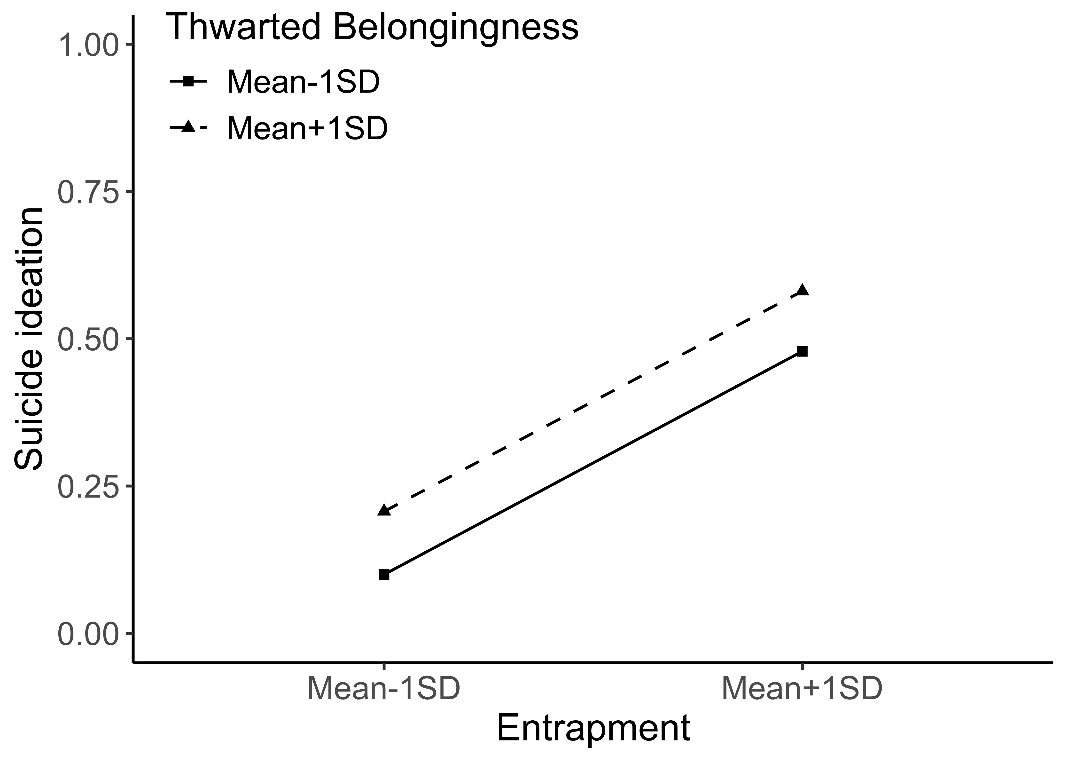 |
| --- | --- |
| \| **Name** \| **Estimate** \| **95% CI** \| \| --- \| --- \| --- \| \| Entrapment \| 1.33*** \| 0.96 to 1.70 \| \| Thwarted Belongingness \| 0.43*** \| 0.23 to 0.62 \| \| Interaction \| -0.44 \| -0.88 to 0.02 \|   R^2^=0.26, AIC=1531 | \| **Name** \| **Estimate** \| **95% CI** \| \| --- \| --- \| --- \| \| Entrapment \| 1.30*** \| 0.92 to 1.67 \| \| Thwarted Belongingness \| 0.43*** \| 0.23 to 0.62 \| \| Interaction \| -0.43 \| -0.87 to 0.03 \| \| **Confounder:** \|  \|  \| \| Sex \| -0.09 \| -0.22 to 0.04 \| \| Age \| -0.0005 \| -0.13 to 0.13 \|   R^2^=0.26, AIC=1533 |

* p<0.05, ** p<0.01, ***p<0.001

**Moderators in path from entrapment to suicide ideation**

| 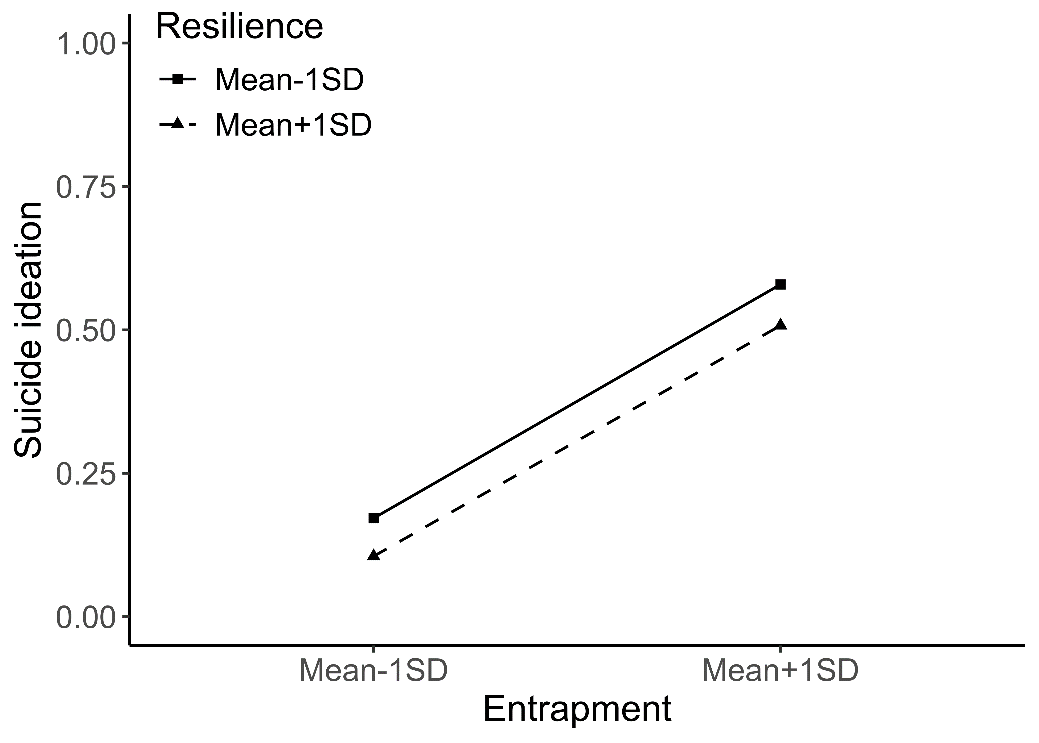 | 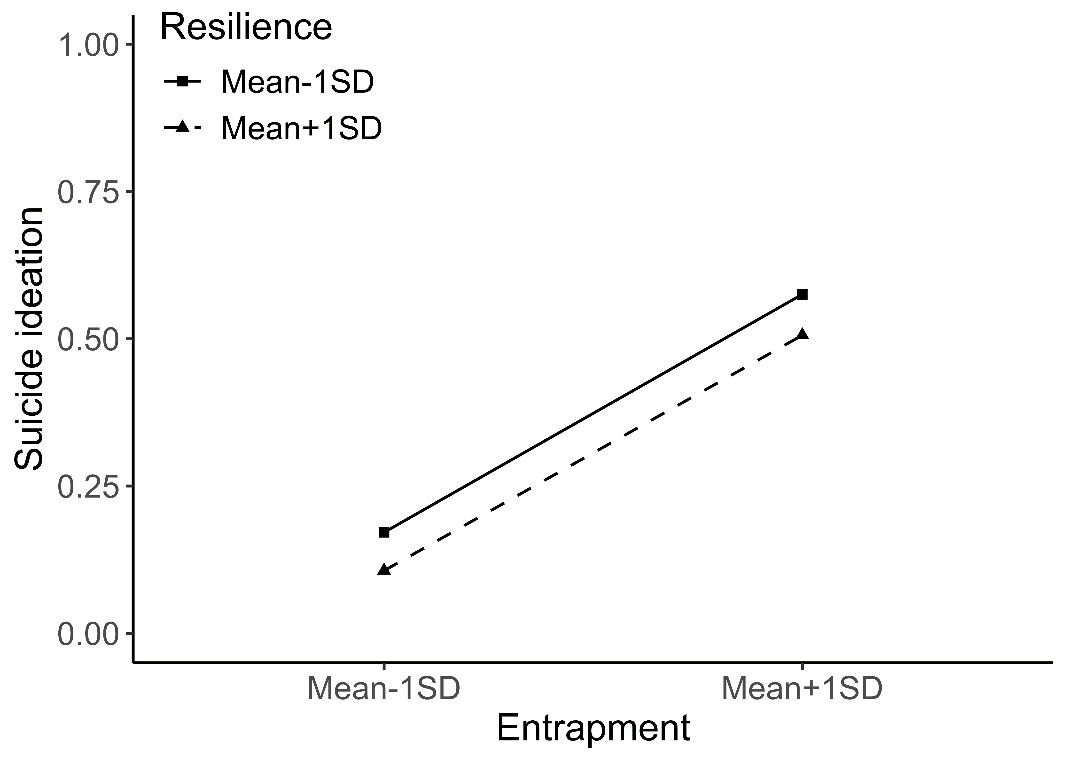 |
| --- | --- |
| \| **Name** \| **Estimate** \| **95% CI** \| \| --- \| --- \| --- \| \| Entrapment \| 0,90*** \| 0,53 to 1,29 \| \| Resilience \| -0,28* \| -0.45 to -0,11 \| \| Interaction \| 0,20 \| -0.15 to 0,54 \|   R^2^=0.26, AIC=1539 | \| **Name** \| **Estimate** \| **95% CI** \| \| --- \| --- \| --- \| \| Entrapment \| 0.89*** \| 0.53 to 1.28 \| \| Resilience \| -0.28* \| -0.45 to -0.10 \| \| Interaction \| 0.19 \| -0.16 to 0.53 \| \| **Confounder:** \|  \|  \| \| Sex \| -0.05 \| -0.18 to 0.08 \| \| Age \| 0.01 \| -0.12 to 0.14 \|   R^2^=0.26, AIC=1543 |

* p<0.05, ** p<0.01, ***p<0.001

**Moderators in path from suicide ideation to intentional self-harm**

| 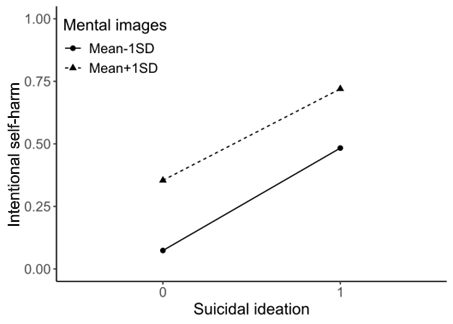 | 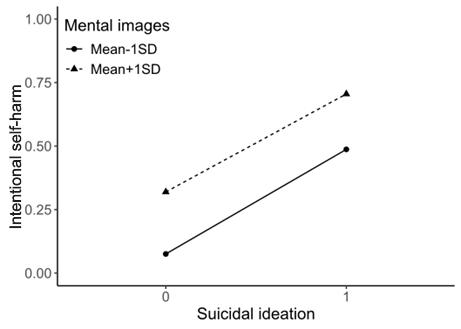 |
| --- | --- |
| \| **Name** \| **Estimate** \| **95% CI** \| \| --- \| --- \| --- \| \| Suicide ideation \| 2.00*** \| 1.72 to 2.29 \| \| Mental images \| 0.97*** \| 0.72 to 1.22 \| \| Interaction \| -0.46** \| -0.77 to -0.15 \|   R^2^=0.33, AIC=1422 | \| **Name** \| **Estimate** \| **95% CI** \| \| --- \| --- \| --- \| \| Suicide ideation \| 2.04*** \| 1.75 to 2.33 \| \| Mental images \| 0.88*** \| 0.63 to 1.13 \| \| Interaction \| -0.41** \| -0.73 to -0.10 \| \| **Confounder:** \|  \|  \| \| Sex \| -0.43*** \| -0.57 to -0.30 \| \| Age \| 0.11 \| -0.03 to 0.24 \|   R^2^=0.35, AIC=1384 |

* p<0.05, ** p<0.01, ***p<0.001

**Moderators in path from suicide ideation to intentional self-harm**

| 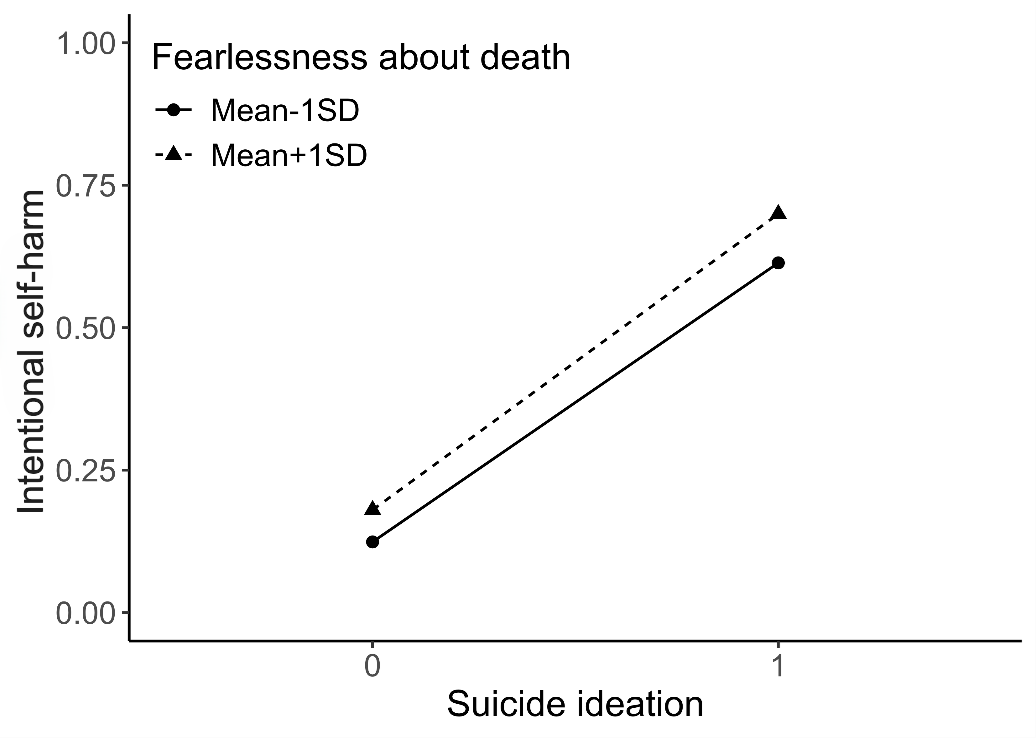 | 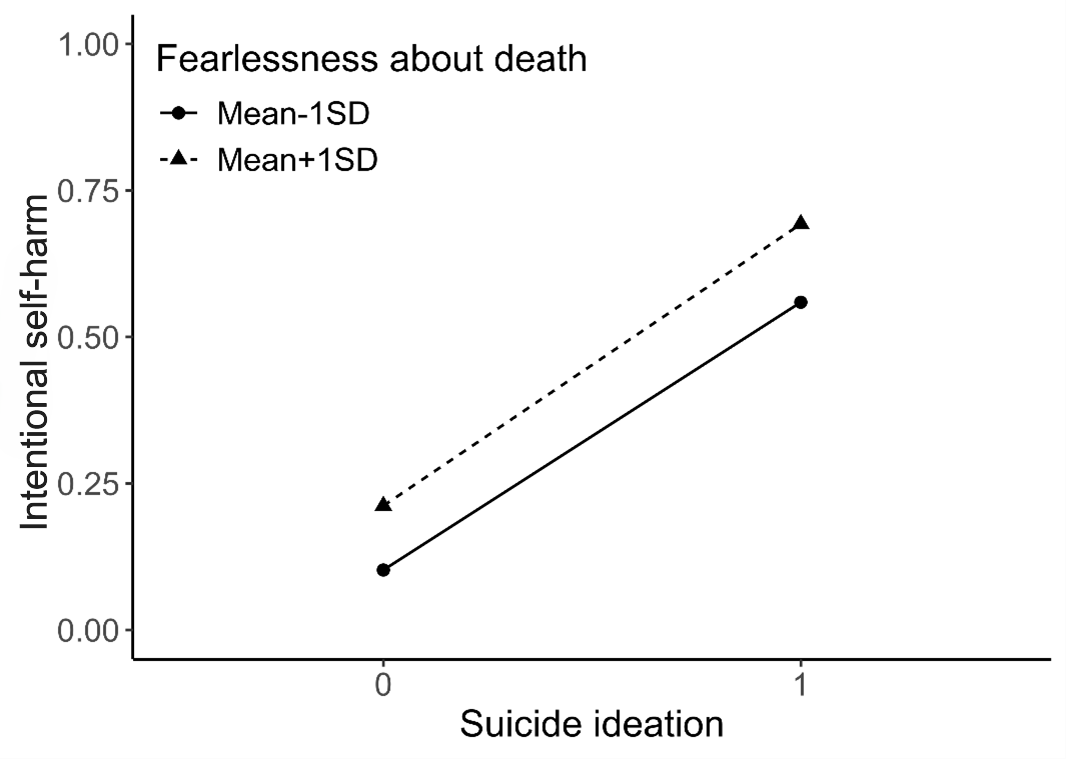 |
| --- | --- |
| \| **Name** \| **Estimate** \| **95% CI** \| \| --- \| --- \| --- \| \| Suicide ideation \| 2.39*** \| 2.12 to 2.67 \| \| Fearlessness about death \| 0.22* \| 0.02 to 0.42 \| \| Interaction \| -0.03 \| -0.30 to 0.24 \|   R^2^=0.29, AIC=1504 | \| **Name** \| **Estimate** \| **95% CI** \| \| --- \| --- \| --- \| \| Suicide ideation \| 2.27*** \| 1.99 to 2.56 \| \| Fearlessness about death \| 0.43*** \| 0.22 to 0.64 \| \| Interaction \| -0.14 \| -0.42 to 0.14 \| \| **Confounder:** \|  \|  \| \| Sex \| -0.58*** \| -0.72 to -0.45 \| \| Age \| 0.13 \| 0.00 to 0.26 \|   R^2^=0.33, AIC=1443 |

* p<0.05, ** p<0.01, ***p<0.001

**Moderators in path from suicide ideation to intentional self-harm**

| 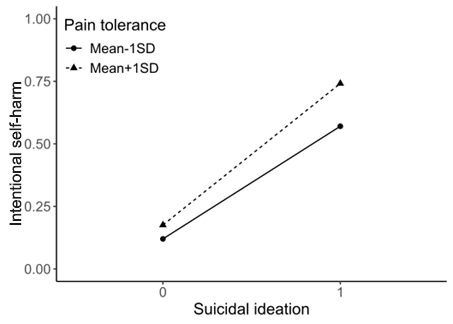 | 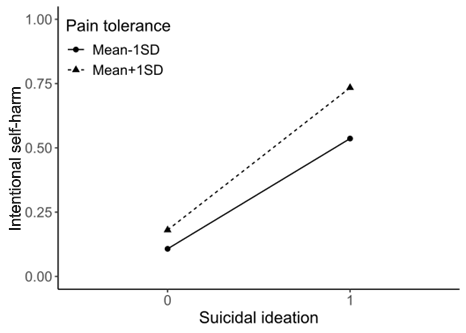 |
| --- | --- |
| \| **Name** \| **Estimate** \| **95% CI** \| \| --- \| --- \| --- \| \| Suicide ideation \| 2.44** \| 2.18 to 2.70 \| \| Pain tolerance \| 0.22** \| 0.05 to 0.39 \| \| Interaction \| 0.16 \| -0.10 to 0.43 \|   R^2^=0.30, AIC=1492 | \| **Name** \| **Estimate** \| **95% CI** \| \| --- \| --- \| --- \| \| Suicide ideation \| 2.40*** \| 2.13 to 2.67 \| \| Pain tolerance \| 0.30** \| 0.13 to 0.48 \| \| Interaction \| 0.13 \| -0.14 to 0.40 \| \| **Confounder:** \|  \|  \| \| Sex \| -0.55*** \| -0.69 to -0.42 \| \| Age \| 0.08 \| -0.05 to 0.21 \|   R^2^=0.33, AIC=1429 |

* p<0.05, ** p<0.01, ***p<0.001

**Moderators in path from suicide ideation to intentional self-harm**

| 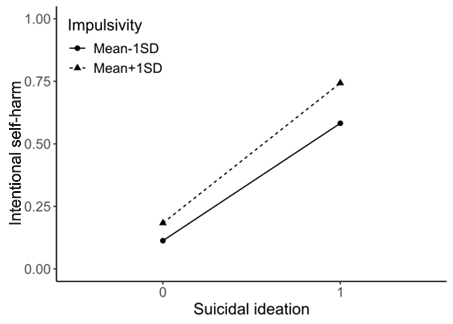 | 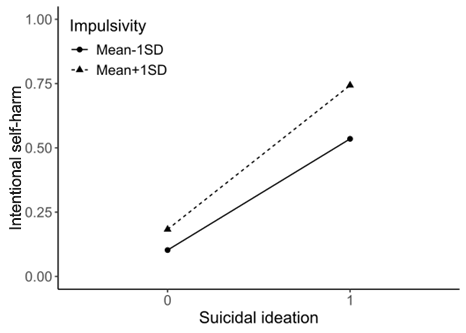 |
| --- | --- |
| \| **Name** \| **Estimate** \| **95% CI** \| \| --- \| --- \| --- \| \| Suicide ideation \| 2.47*** \| 2.22 to 2.74 \| \| Impulsivity \| 0.28** \| 0.11 to 0.45 \| \| Interaction \| 0.08 \| -0.18 to 0.34 \|   R^2^=0.30, AIC=1488 | \| **Name** \| **Estimate** \| **95% CI** \| \| --- \| --- \| --- \| \| Suicide ideation \| 2.43*** \| 2.17 to 2.71 \| \| Impulsivity \| 0.34*** \| 0.16 to 0.51 \| \| Interaction \| 0.12 \| -0.14 to 0.39 \| \| **Confounder:** \|  \|  \| \| Sex \| -0.56*** \| -0.70 to -0.43 \| \| Age \| 0.10 \| -0.04 to 0.23 \|   R^2^=0.34, AIC=1422 |

* p<0.05, ** p<0.01, ***p<0.001

**Moderators in path from suicide ideation to intentional self-harm**

| 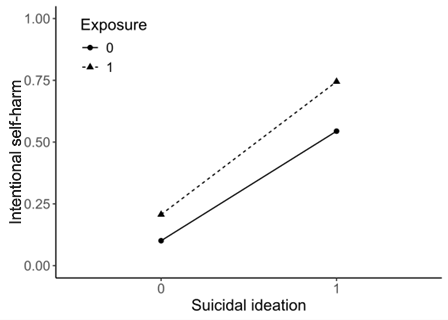 | 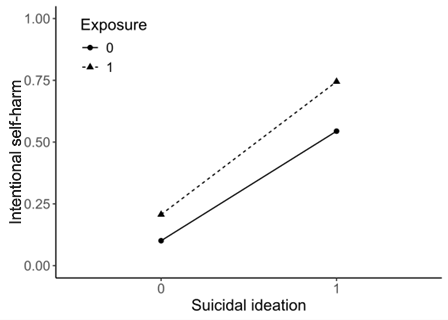 |
| --- | --- |
| \| **Name** \| **Estimate** \| **95% CI** \| \| --- \| --- \| --- \| \| Suicide ideation \| 2.37*** \| 1.96 to 2.78 \| \| Exposure \| 0.84*** \| 0.50 to 1.19 \| \| Interaction \| 0.05 \| -0.48 to 0.58 \|   R^2^=0.31, AIC=1471 | \| **Name** \| **Estimate** \| **95% CI** \| \| --- \| --- \| --- \| \| Suicide ideation \| 2.34*** \| 1.93 to 2.77 \| \| Exposure \| 0.59** \| 0.24 to 0.95 \| \| Interaction \| 0.12 \| -0.42 to 0.66 \| \| **Confounder:** \|  \|  \| \| Sex \| -0.43*** \| -0.57 to -0.29 \| \| Age \| 0.04 \| -0.09 to 0.18 \|   R^2^=0.33, AIC=1436 |

* p<0.05, ** p<0.01, ***p<0.001

**Moderators in path from suicide ideation to non-suicidal self-injury**

| 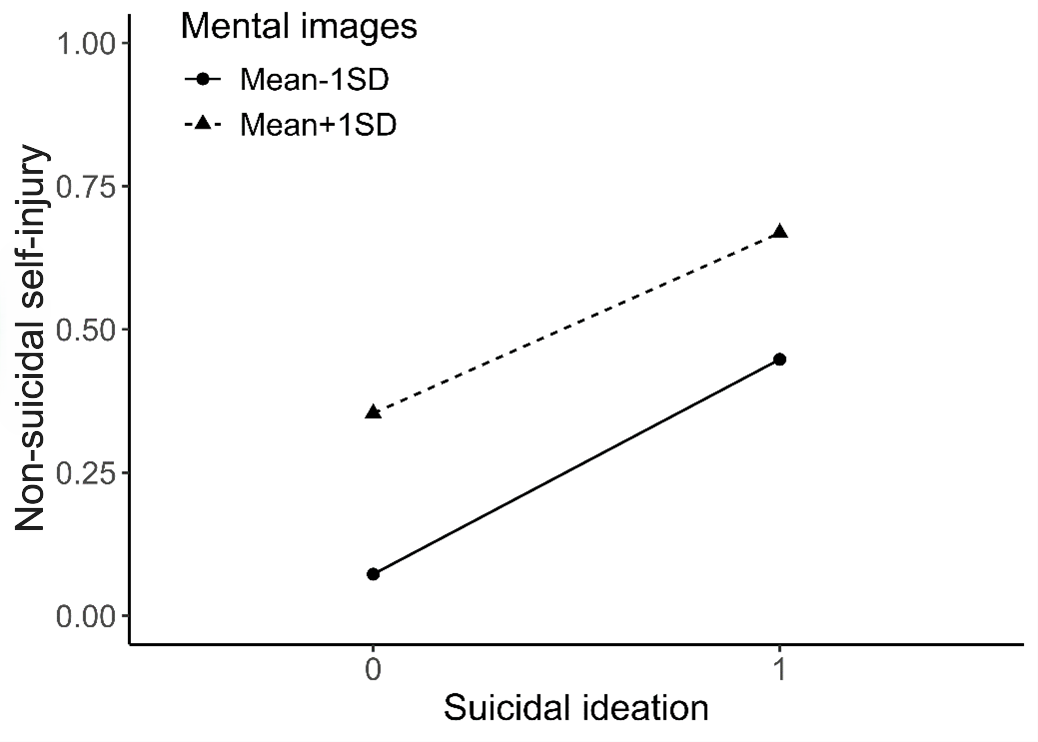 | 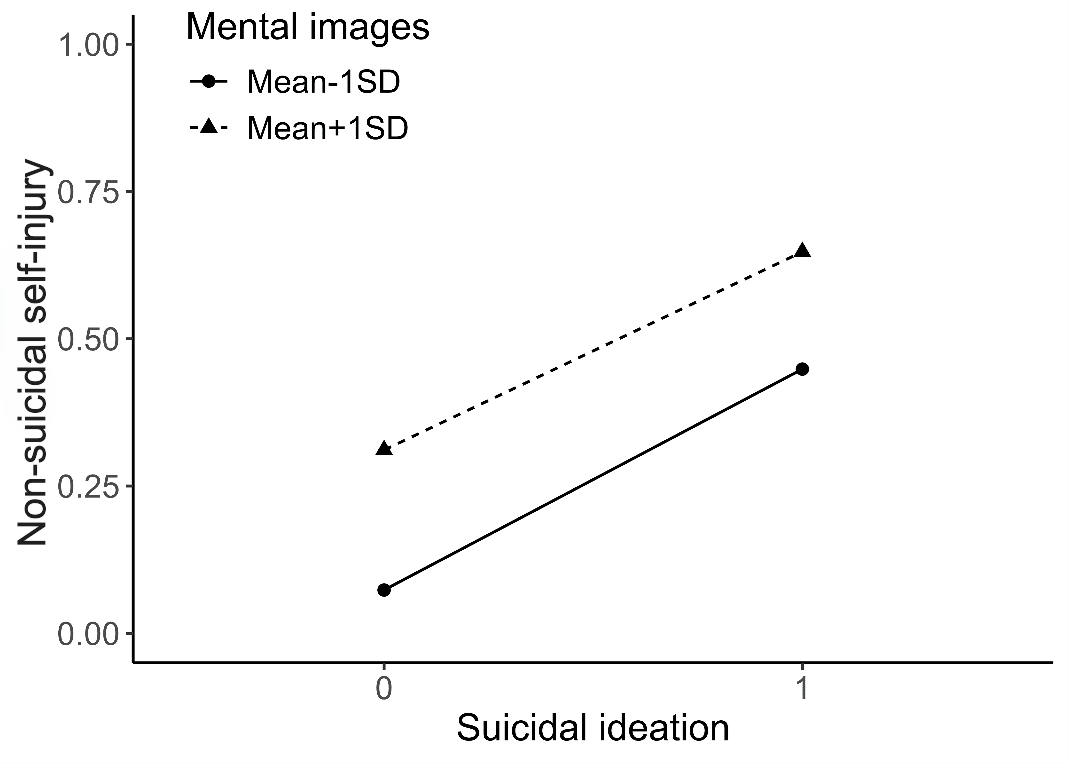 |
| --- | --- |
| \| **Name** \| **Estimate** \| **95% CI** \| \| --- \| --- \| --- \| \| Suicide ideation \| 1,82*** \| 1,54 to 2,10 \| \| Mental images \| 0,97*** \| 0.72 to 1,22 \| \| Interaction \| -0.51** \| -0,82 to -0,21 \|   R^2^=0.30, AIC=1450 | \| **Name** \| **Estimate** \| **95% CI** \| \| --- \| --- \| --- \| \| Suicide ideation \| 1.86*** \| 1.58 to 2.16 \| \| Mental images \| 0.87*** \| 0.62 to 1.13 \| \| Interaction \| -0.46*** \| -0.77 to -0.15 \| \| **Confounder:** \|  \|  \| \| Sex \| -0.52*** \| -0.66 to -0.38 \| \| Age \| 0.10 \| -0.03 to 0.24 \|   R^2^=0.33, AIC=1396 |

* p<0.05, ** p<0.01, ***p<0.001

**Moderators in path from suicide ideation to non-suicidal self-injury**

| 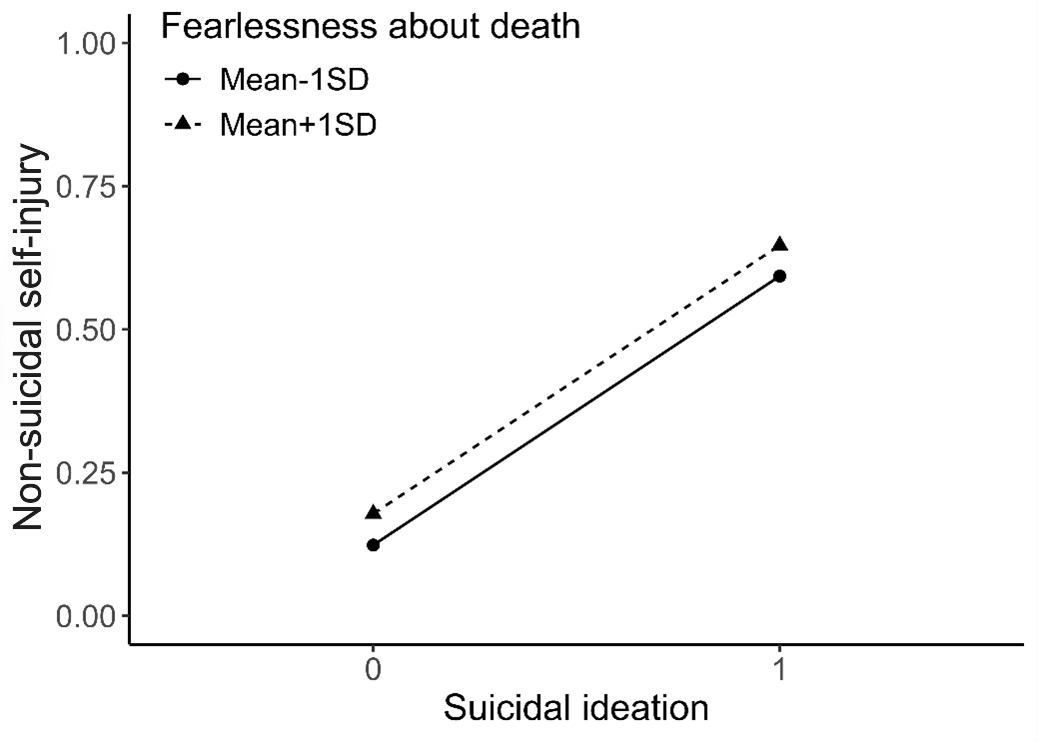 | 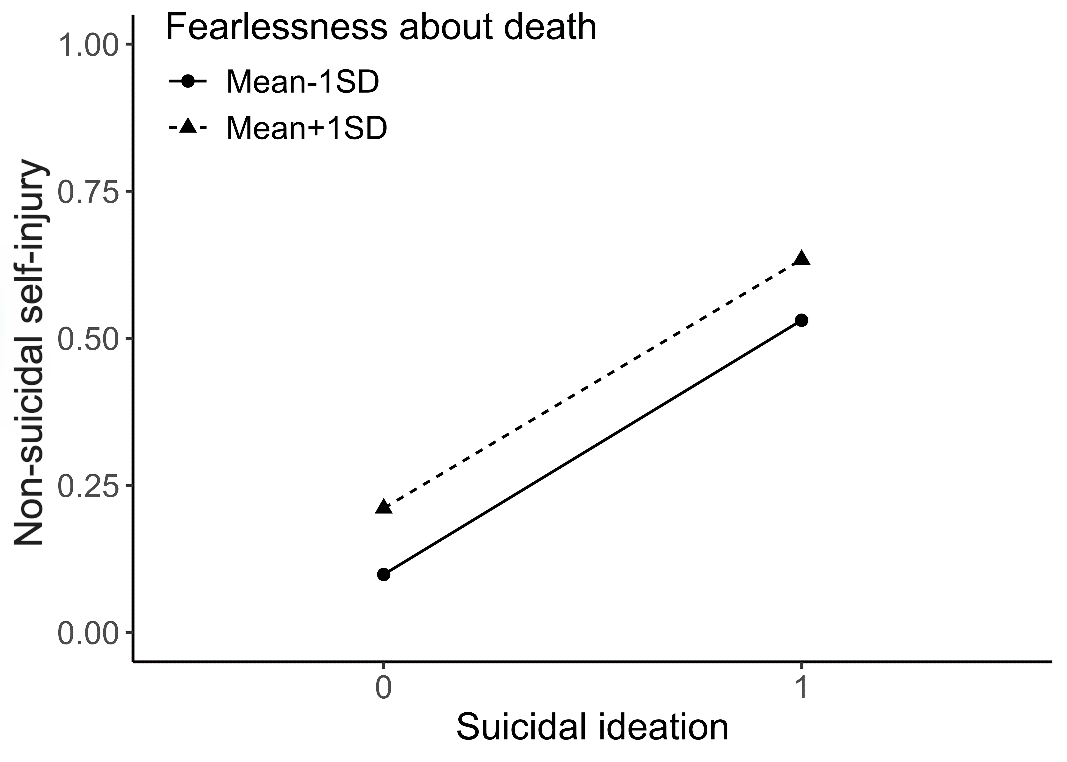 |
| --- | --- |
| \| **Name** \| **Estimate** \| **95% CI** \| \| --- \| --- \| --- \| \| Suicide ideation \| 2.23*** \| 1,96 to 2,51 \| \| Fearlessness about death \| 0.22* \| 0,01 to 0,42 \| \| Interaction \| -0.10 \| -0,37 to 0,16 \|   R^2^=0.25, AIC=1533 | \| **Name** \| **Estimate** \| **95% CI** \| \| --- \| --- \| --- \| \| Suicide ideation \| 2.10*** \| 1,82 to 2,39 \| \| Fearlessness about death \| 0.45*** \| 0.23 to 0.66 \| \| Interaction \| -0.23 \| -0.51 to 0.04 \| \| **Confounder:** \|  \|  \| \| Sex \| -0.66*** \| -0.80 to -0.52 \| \| Age \| 0.12 \| -0.01 to 0,25 \|   R^2^=0.30, AIC=1442 |

* p<0.05, ** p<0.01, ***p<0.001

**Moderators in path from suicide ideation to non-suicidal self-injury**

| 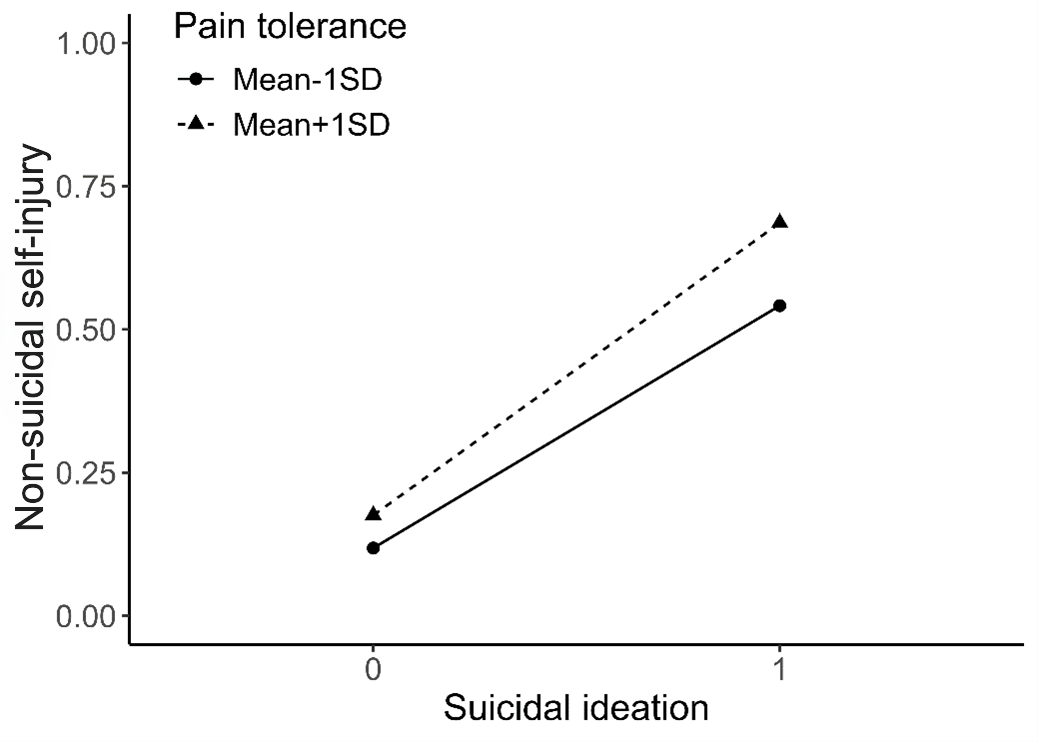 | 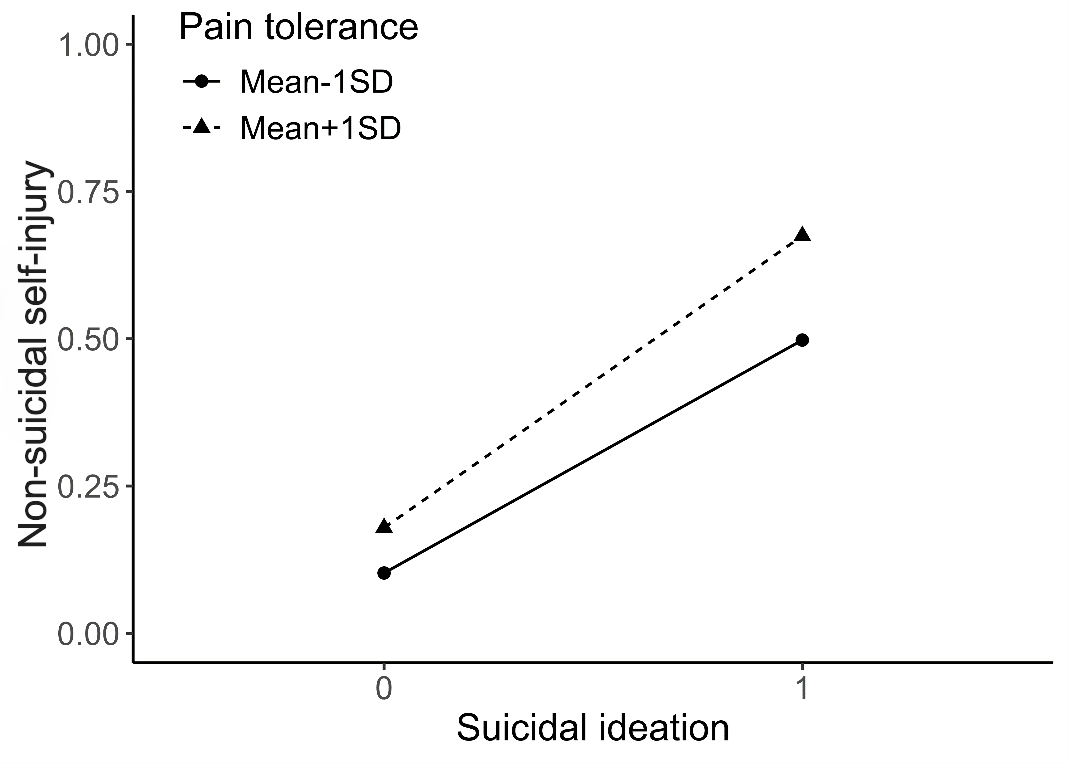 |
| --- | --- |
| \| **Name** \| **Estimate** \| **95% CI** \| \| --- \| --- \| --- \| \| Suicide ideation \| 2.25*** \| 1.99 to 2,51 \| \| Pain tolerance \| 0.23* \| 0,06 to 0,40 \| \| Interaction \| 0.08 \| -0,18 to 0,34 \|   R^2^=0.26, AIC=1521 | \| **Name** \| **Estimate** \| **95% CI** \| \| --- \| --- \| --- \| \| Suicide ideation \| 2.21*** \| 1,94 to 2,48 \| \| Pain tolerance \| 0,32** \| 0.15 to 0.50 \| \| Interaction \| 0,04 \| -0.22 to 0,31 \| \| **Confounder:** \|  \|  \| \| Sex \| -0,63*** \| -0.77 to -0.49 \| \| Age \| 0.07 \| -0.06 to 0.20 \|   R^2^=0.30, AIC=1438 |

* p<0.05, ** p<0.01, ***p<0.001

**Moderators in path from suicide ideation to non-suicidal self-injury**

| 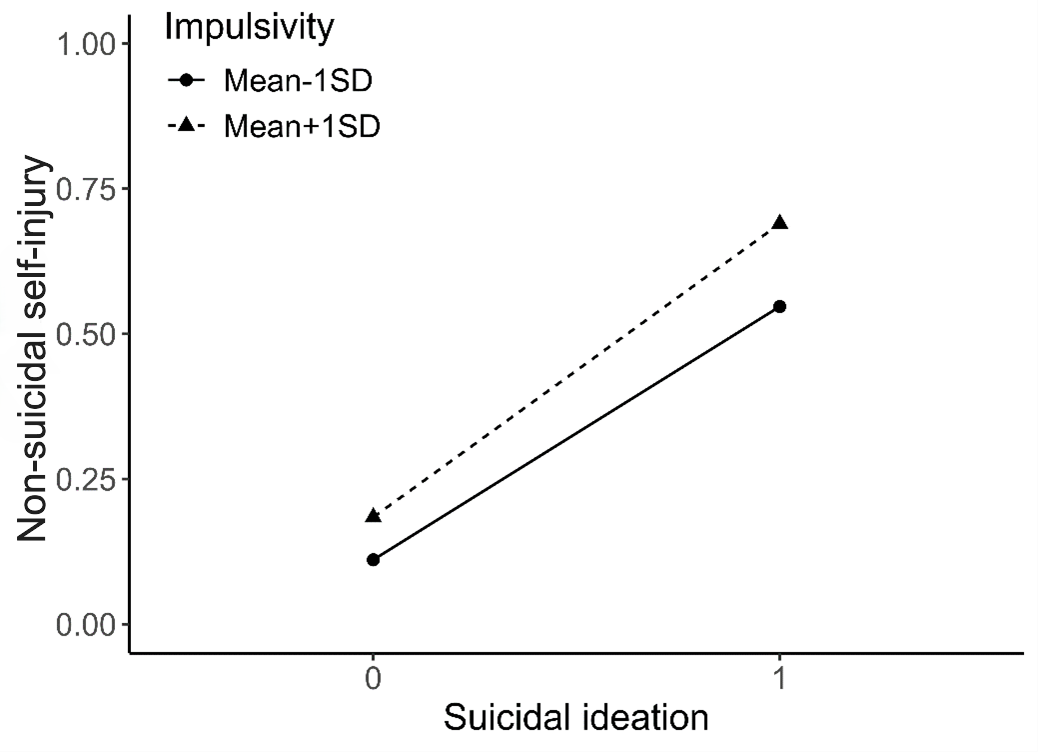 | 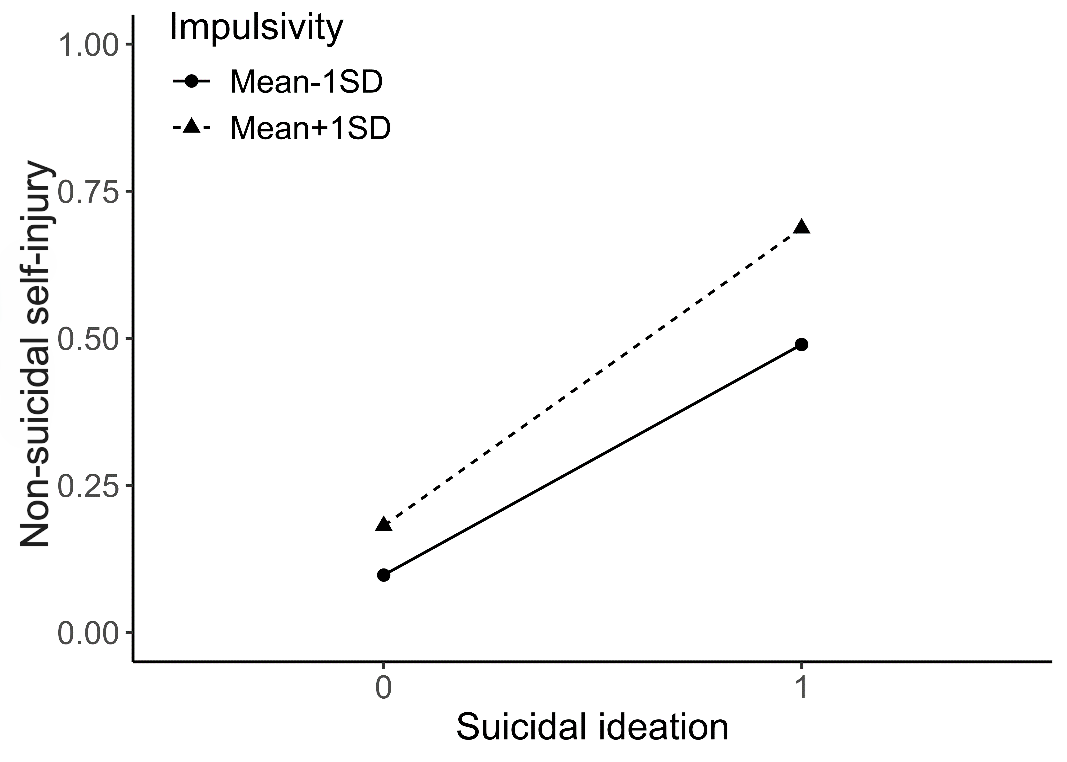 |
| --- | --- |
| \| **Name** \| **Estimate** \| **95% CI** \| \| --- \| --- \| --- \| \| Suicide ideation \| 2.28*** \| 2.02 to 2,54 \| \| Impulsivity \| 0.30** \| 0,12 to 0,47 \| \| Interaction \| 0.01 \| -0.24 to 0,26 \|   R^2^=0.26, AIC=1517 | \| **Name** \| **Estimate** \| **95% CI** \| \| --- \| --- \| --- \| \| Suicide ideation \| 2.24*** \| 1,97 to 2,51 \| \| Impulsivity \| 0,36*** \| 0,18 to 0,53 \| \| Interaction \| 0,06 \| -0,20 to 0,32 \| \| **Confounder:** \|  \|  \| \| Sex \| -0,64*** \| -0,78 to -0,50 \| \| Age \| 0,09 \| -0,04 to 0,22 \|   R^2^=0.31, AIC=1430 |

* p<0.05, ** p<0.01, ***p<0.001

**Moderators in path from suicide ideation to non-suicidal self-injury**

| 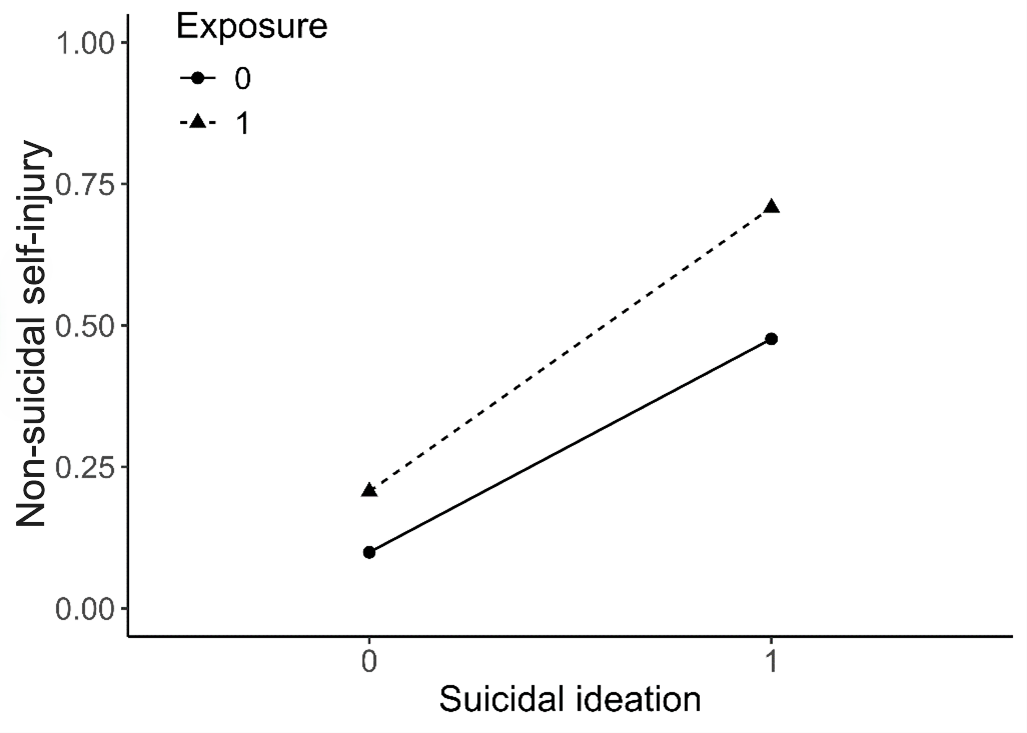 | 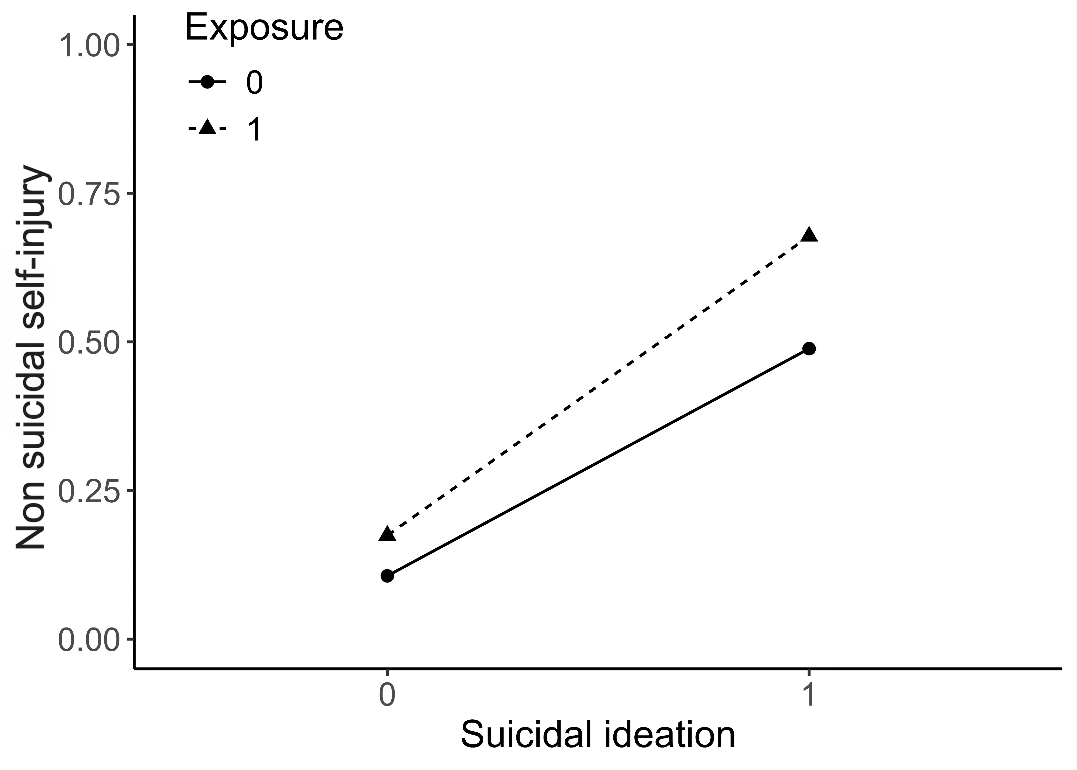 |
| --- | --- |
| \| **Name** \| **Estimate** \| **95% CI** \| \| --- \| --- \| --- \| \| Suicide ideation \| 2.11*** \| 1.70 to 2.53 \| \| Exposure \| 0.86*** \| 0.52 to 1.21 \| \| Interaction \| 0.12 \| -0.41 to 0.65 \|   R^2^=0.27, AIC=1491 | \| **Name** \| **Estimate** \| **95% CI** \| \| --- \| --- \| --- \| \| Suicide ideation \| 2,08*** \| 1,66 to 2,51 \| \| Exposure \| 0,57** \| 0,22 to 0,93 \| \| Interaction \| 0,22 \| -0,33 to 0,76 \| \| **Confounder:** \|  \|  \| \| Sex \| -0,51*** \| -0,65 to -0,37 \| \| Age \| 0,04 \| -0,09 to 0,17 \|   R^2^=0.30, AIC=1441 |

* p<0.05, ** p<0.01, ***p<0.001

**Moderators in path from suicide ideation to suicide attempt**

| 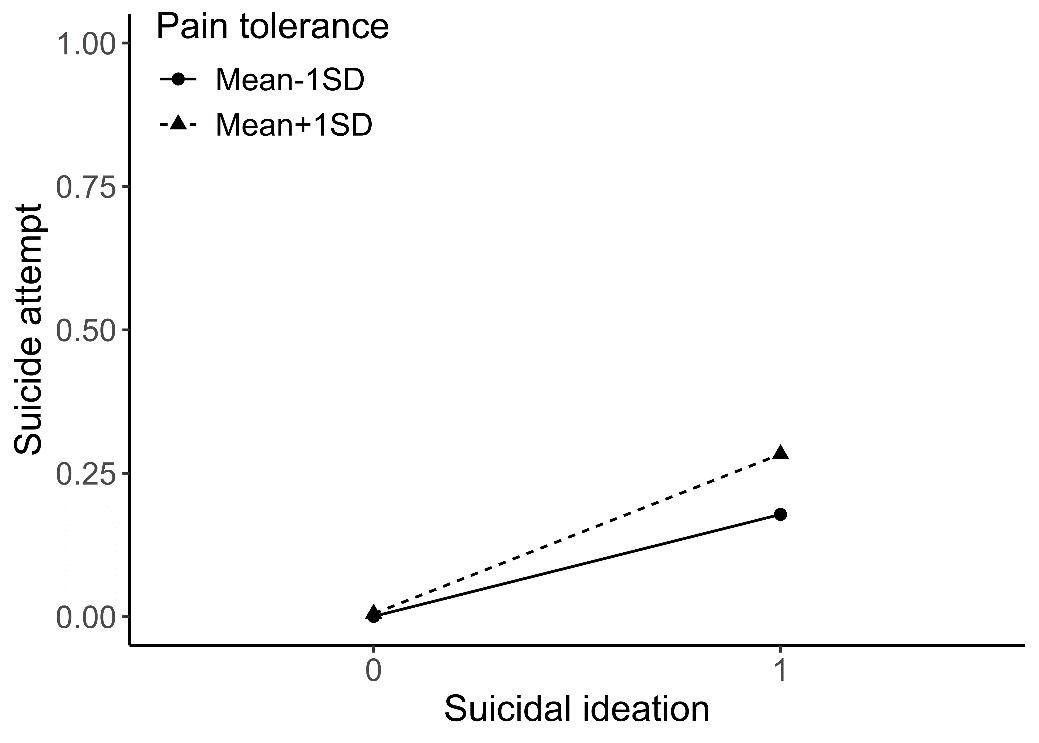 | 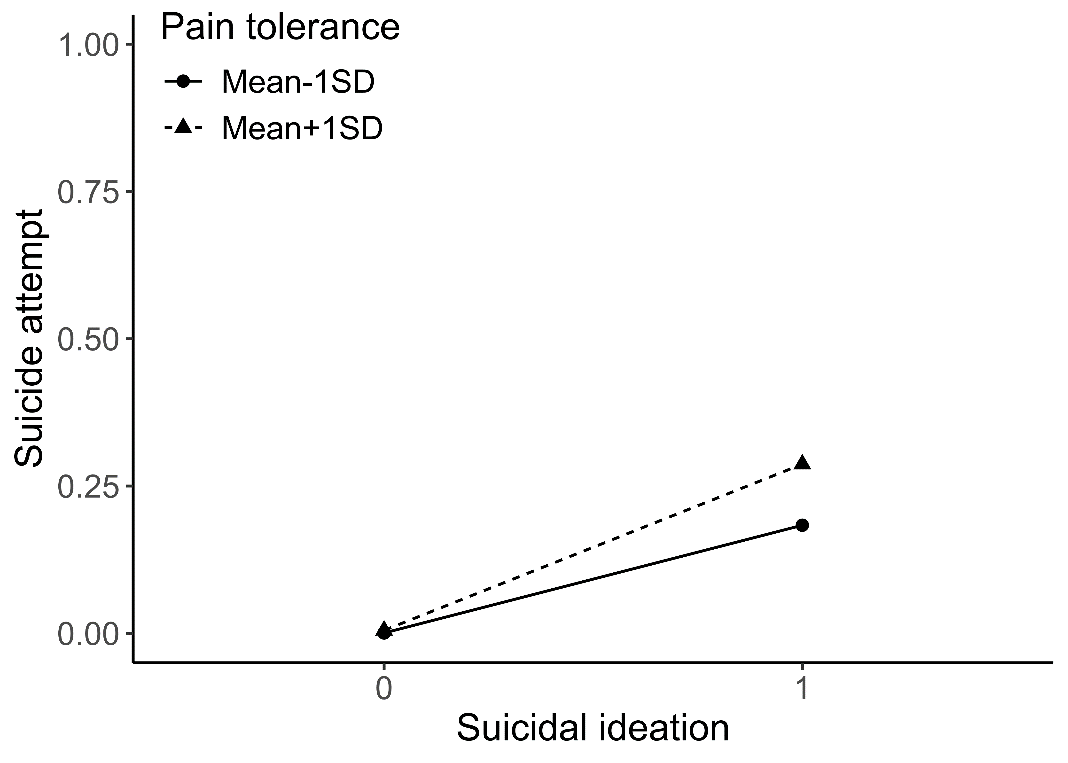 |
| --- | --- |
| \| **Name** \| **Estimate** \| **95% CI** \| \| --- \| --- \| --- \| \| Suicide ideation \| 5,14** \| 3,87 to 7,46 \| \| Pain tolerance \| 1,12 \| -0,05 to 2,65 \| \| Interaction \| -0,82 \| -2,36 to 0,38 \|   R^2^=0.19, AIC=572 | \| **Name** \| **Estimate** \| **95% CI** \| \| --- \| --- \| --- \| \| Suicide ideation \| 5.19** \| 3.91 to 7.51 \| \| Pain tolerance \| 1.10 \| -0.07 to 2.62 \| \| Interaction \| -0.81 \| -2.34 to 0.39 \| \| **Confounder:** \|  \|  \| \| Sex \| 0.14 \| -0.08 to 0.35 \| \| Age \| -0.04 \| -0.25 to 0.17 \|   R^2^=0.19, AIC=574 |

* p<0.05, ** p<0.01, ***p<0.001

**Moderators in path from suicide ideation to suicide attempt**

| 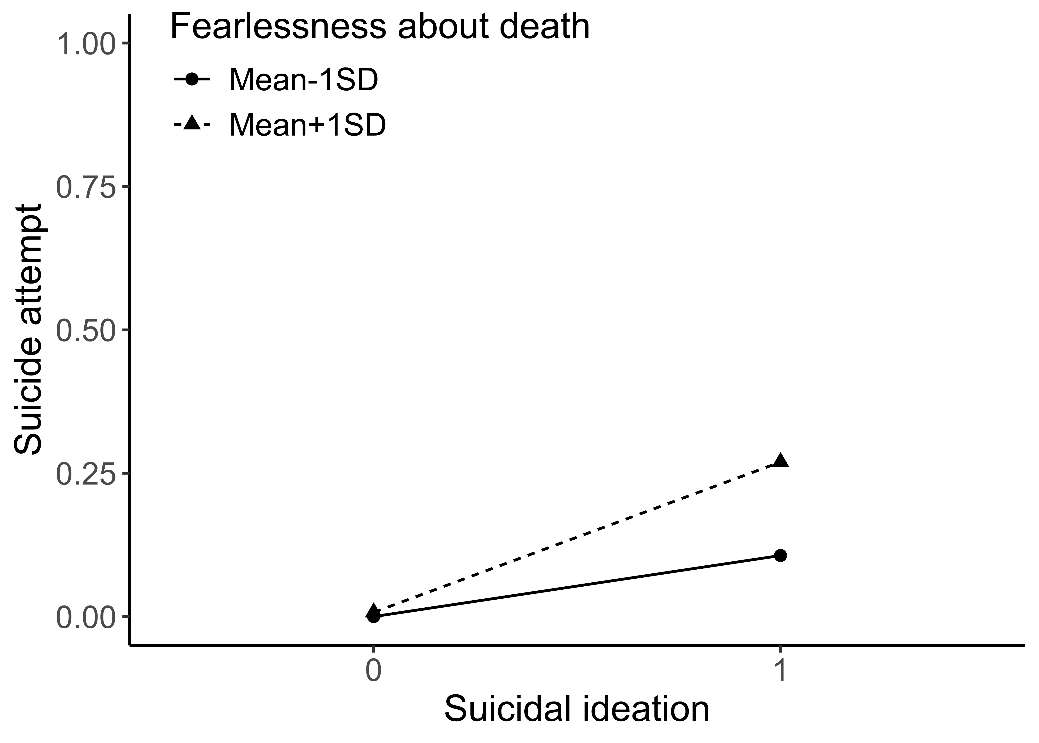 | 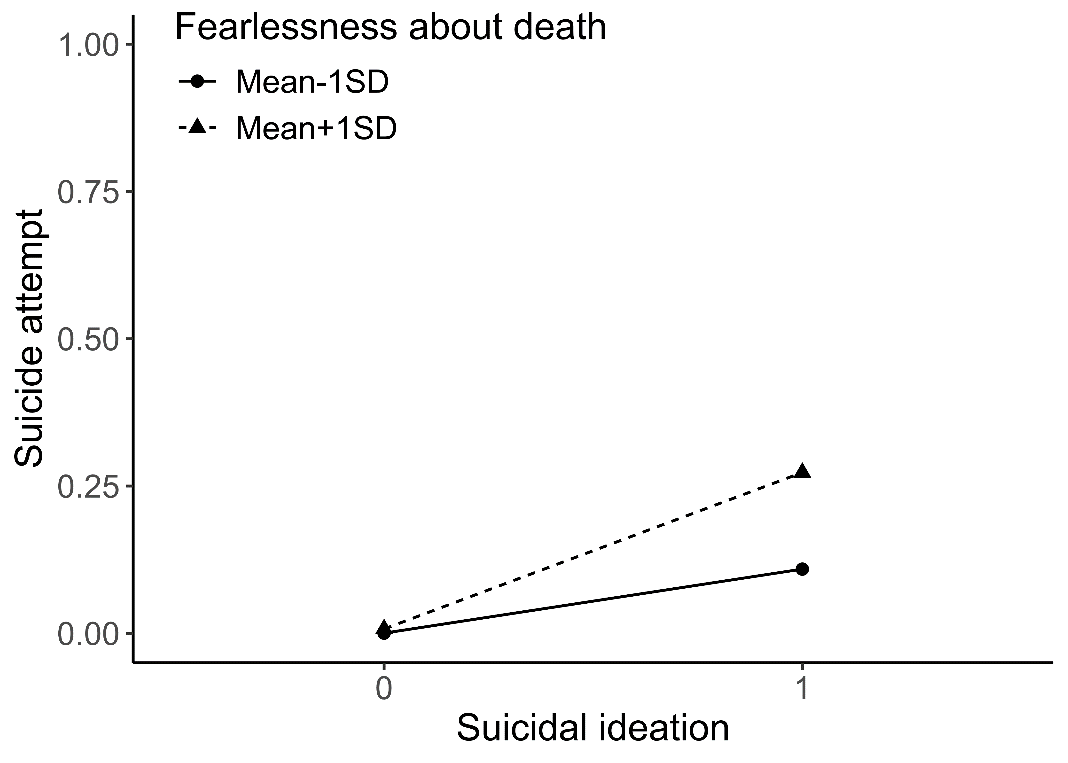 |
| --- | --- |
| \| **Name** \| **Estimate** \| **95% CI** \| \| --- \| --- \| --- \| \| Suicide ideation \| 4,97*** \| 3,59 to 7.26 \| \| Fearlessness about death \| 1,67* \| 0,49 to 2,94 \| \| Interaction \| -1,10 \| -2,39 to 0,09 \|   R^2^=0.23, AIC=543 | \| **Name** \| **Estimate** \| **95% CI** \| \| --- \| --- \| --- \| \| Suicide ideation \| 5.01*** \| 3.63 to 7.31 \| \| Fearlessness about death \| 1.65* \| 0.47 to 2.92 \| \| Interaction \| -1.09 \| -2.38 to 0.11 \| \| **Confounder:** \|  \|  \| \| Sex \| 0.09 \| -0.13 to 0.30 \| \| Age \| 0.03 \| -0.18 to 0.24 \|   R^2^=0.24, AIC=546 |

* p<0.05, ** p<0.01, ***p<0.001

**Moderators in path from suicide ideation to suicide attempt**

| 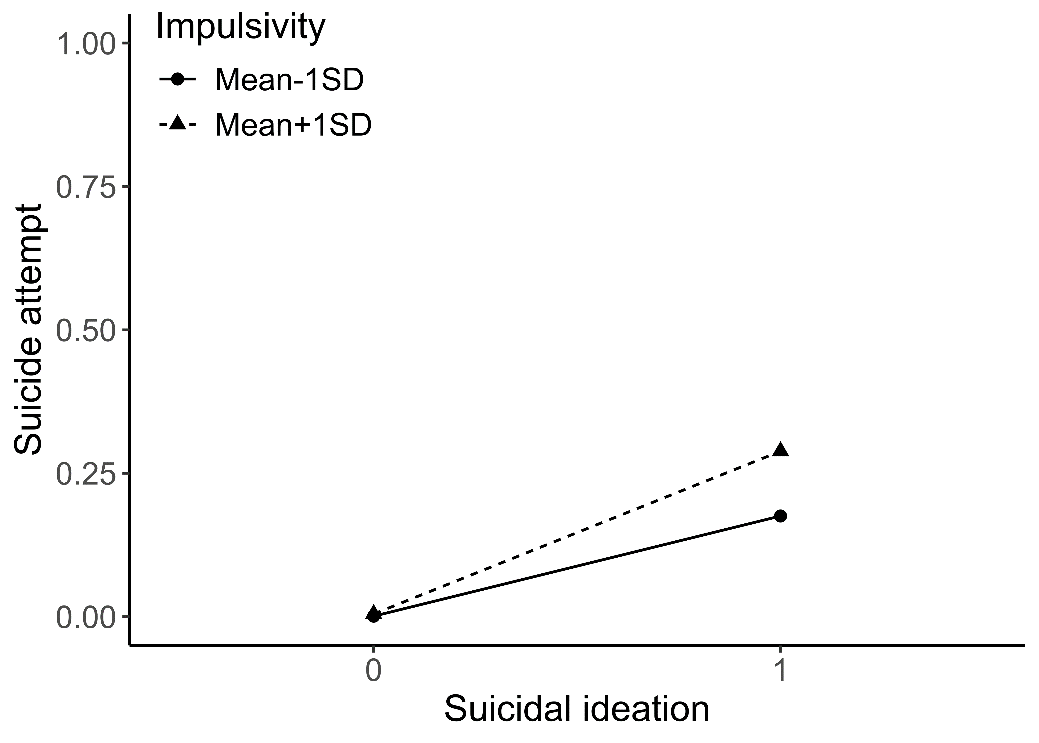 | 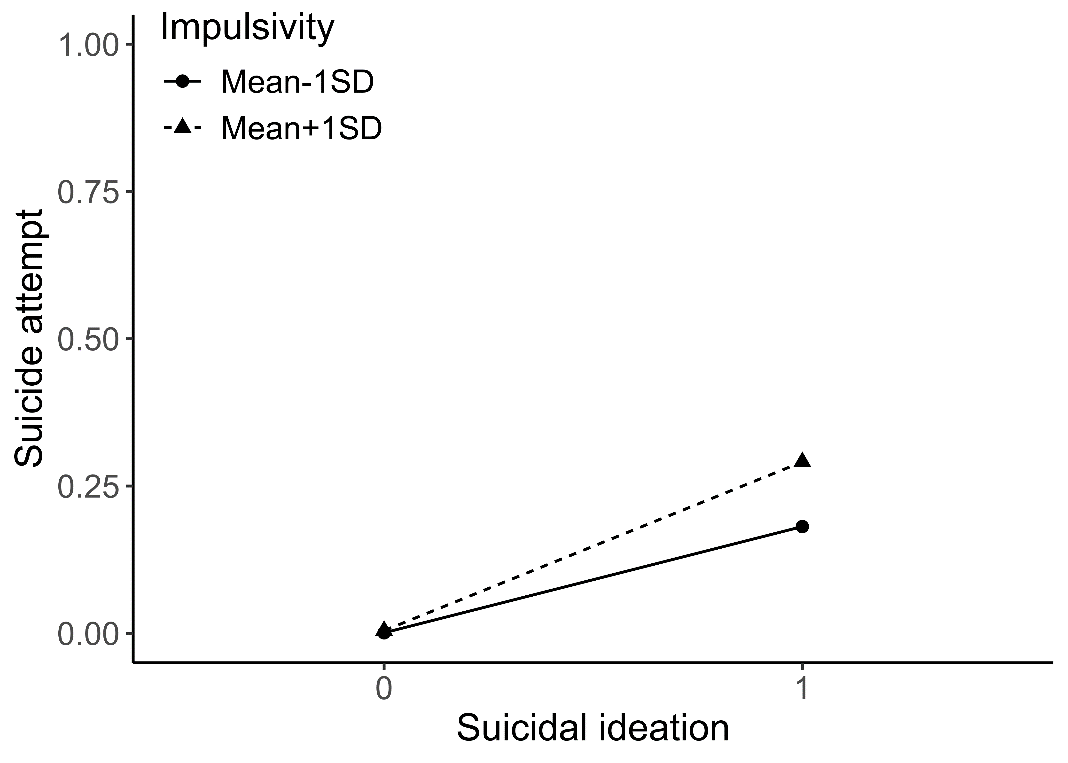 |
| --- | --- |
| \| **Name** \| **Estimate** \| **95% CI** \| \| --- \| --- \| --- \| \| Suicide ideation \| 4,93** \| 3,80 to 6,72 \| \| Impulsivity \| 0,81 \| -0,25 to 1,79 \| \| Interaction \| -0,48 \| -1,48 to 0,59 \|   R^2^=0.20, AIC=570 | \| **Name** \| **Estimate** \| **95% CI** \| \| --- \| --- \| --- \| \| Suicide ideation \| 4.97** \| 3.84 to 6.77 \| \| Impulsivity \| 0.80 \| -0.26 to 1.79 \| \| Interaction \| -0.49 \| -1.50 to 0.58 \| \| **Confounder:** \|  \|  \| \| Sex \| 0.12 \| -0.10 to 0.33 \| \| Age \| -0.02 \| -0.23 to 0.19 \|   R^2^=0.20, AIC=573 |

* p<0.05, ** p<0.01, ***p<0.001

**Moderators in path from suicide ideation to suicide attempt**

| 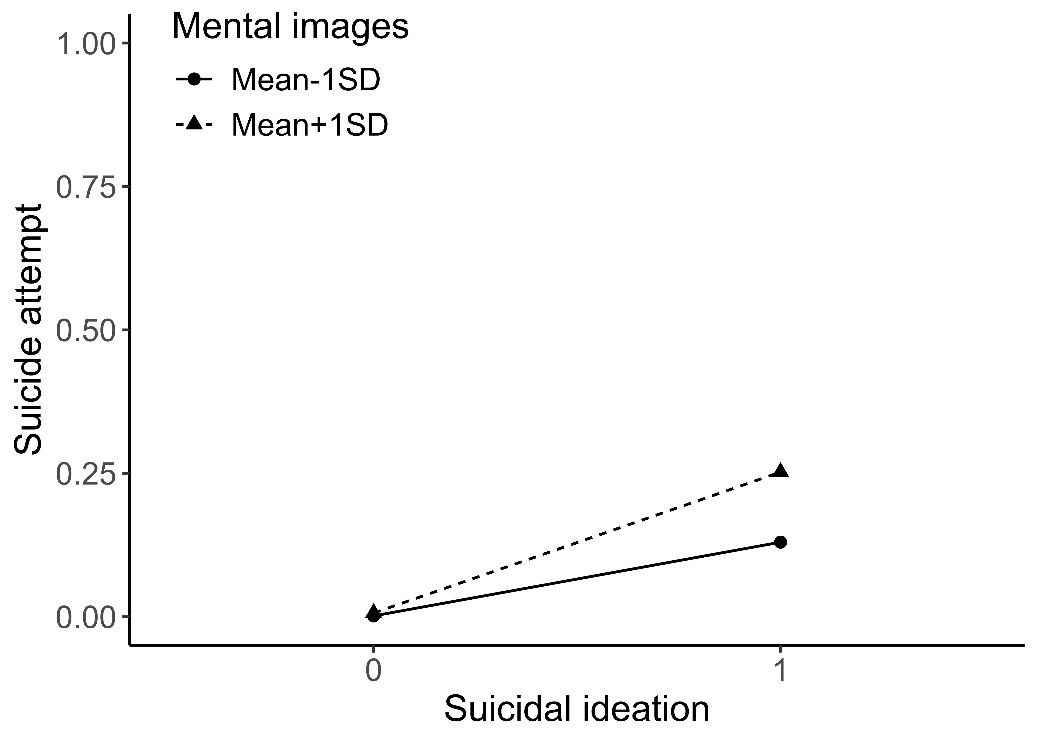 | 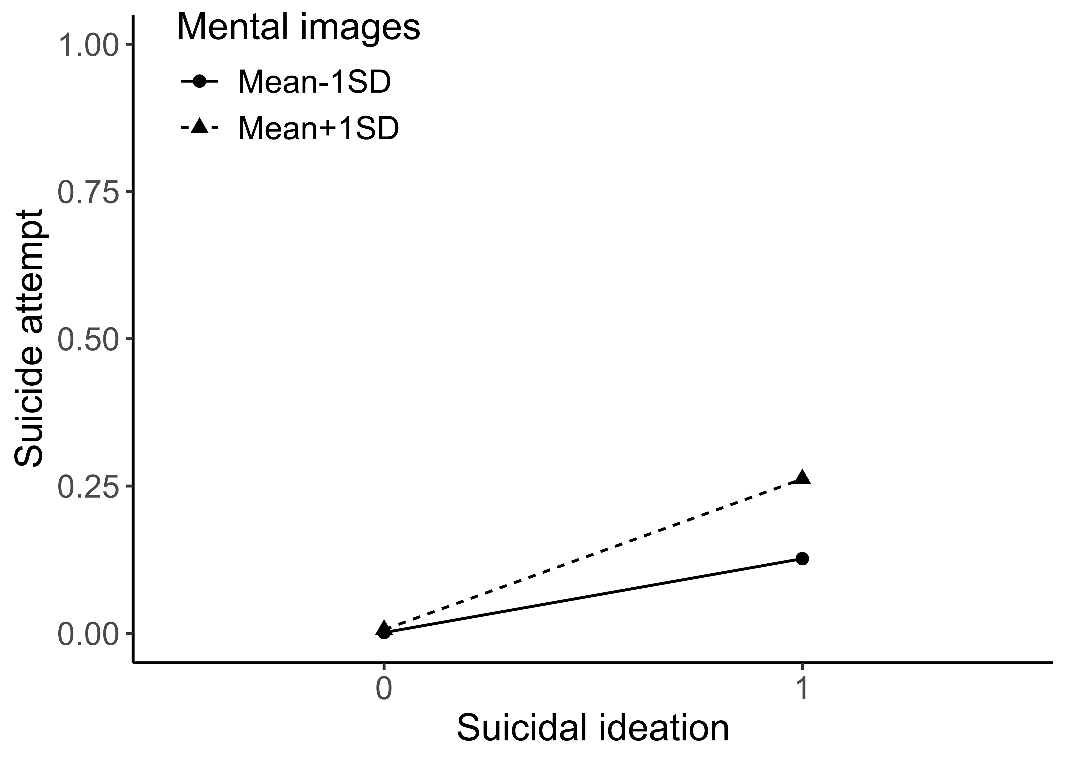 |
| --- | --- |
| \| **Name** \| **Estimate** \| **95% CI** \| \| --- \| --- \| --- \| \| Suicide ideation \| 4,30*** \| 3,29 to 5,73 \| \| Mental images \| 0.76 \| -0,71 to 1,64 \| \| Interaction \| -0.35 \| -1,25 to 1,12 \|   R^2^=0.22, AIC=557 | \| **Name** \| **Estimate** \| **95% CI** \| \| --- \| --- \| --- \| \| Suicide ideation \| 4.35*** \| 3.34 to 5.79 \| \| Mental images \| 0.78 \| -0.66 to 1.63 \| \| Interaction \| -0.33 \| -1.20 to 1.11 \| \| **Confounder:** \|  \|  \| \| Sex \| 0.27* \| 0.05 to 0.50 \| \| Age \| 0.01 \| -0.20 to 0.22 \|   R^2^=0.23, AIC=555 |

* p<0.05, ** p<0.01, ***p<0.001

**Moderators in path from suicide ideation to suicide attempt**

| 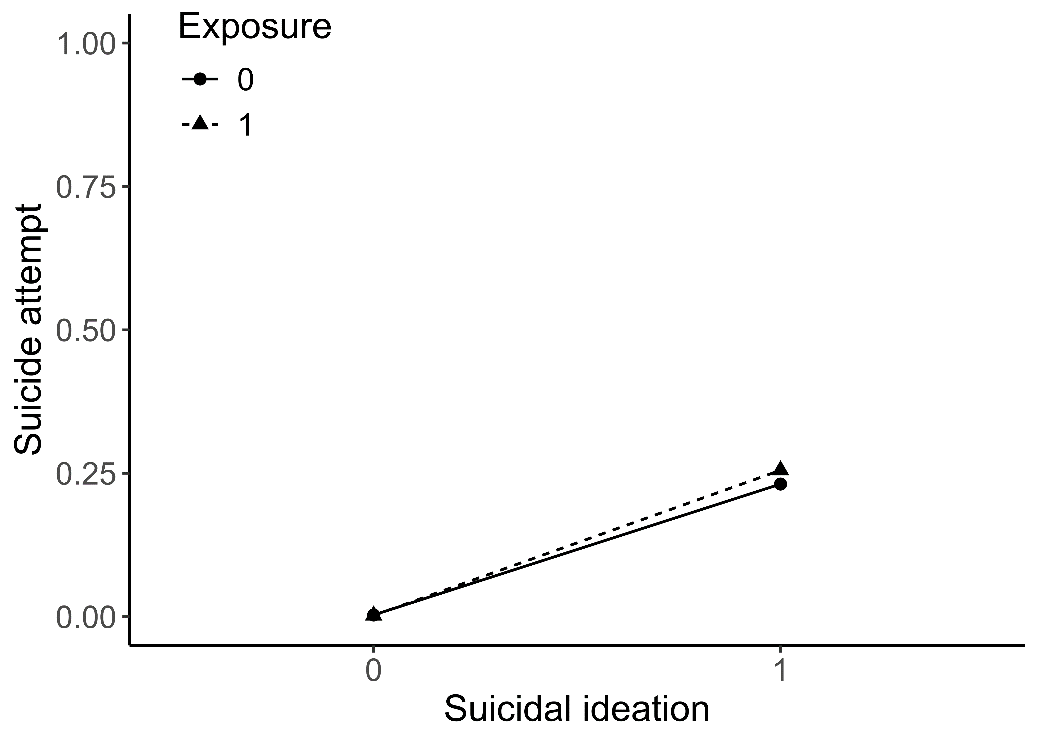 | 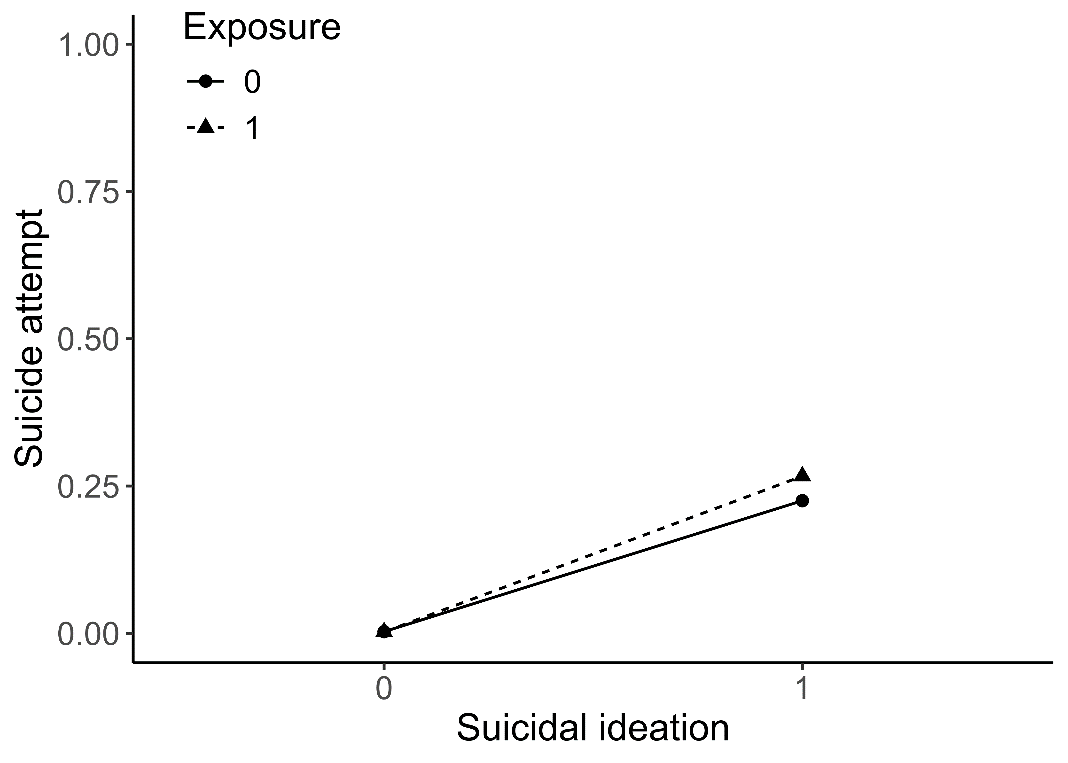 |
| --- | --- |
| \| **Name** \| **Estimate** \| **95% CI** \| \| --- \| --- \| --- \| \| Suicide ideation \| 4.59*** \| 3.38 to 6.41 \| \| Exposure \| -0.32 \| -3,39 to 2,03 \| \| Interaction \| 0.45 \| -1.94 to 3.55 \|   R^2^=0.18, AIC=582 | \| **Name** \| **Estimate** \| **95% CI** \| \| --- \| --- \| --- \| \| Suicide ideation \| 4.63*** \| 3.42 to 6.46 \| \| Exposure \| -0.20 \| -3.27 to 2.16 \| \| Interaction \| 0.42 \| -1.97 to 3.52 \| \| **Confounder:** \|  \|  \| \| Sex \| 0.18 \| -0.04 to 0.39 \| \| Age \| -0.04 \| -1.97 to 3.52 \|   R^2^=0.18, AIC=583 |

* p<0.05, ** p<0.01, ***p<0.001
